# A proteogenomic RNA processing mechanism drives sex differences in meningioma

**DOI:** 10.21203/rs.3.rs-9675139/v1

**Published:** 2026-06-02

**Authors:** David Raleigh, Martha Cady, Ayush Aggarwal, Nicholas Stevers, Naomi Zakimi, Isabelle Liu, Kanish Mirchia, John Coukos, Pornparn Kongpracha, Yuan Zhou, Minh Nguyen, Brooke Braman, Nathan Leclair, Joanna Phillips, Kingsley Chow, Siddarth Shamdasani-Sen, Nefeli Chantousti, Bennedict Choi, Chibo Hong, Ryan Toedebusch, Ryan Englander, Julia Frankus, Diya Sinha, Heather Karner, Jennifer Rosenbluth, Laura Van ′T Veer, Abrar Choudhury, S. John Liu, Nevan Krogan, Joseph Costello, Olga Anczuków, Christine Toedebusch, Pete Dickinson, Hani Goodarzi, Danielle Swaney

**Affiliations:** University of California San Francisco; University of California, San Francisco; University of California San Francisco; University of California San Francisco; University of California San Francisco; University of California, San Francisco; University of California San Francisco; University of California San Francisco; University of California San Francisco; University of California San Francisco; University of California San Francisco; University of California San Francisco; Memorial Sloan Kettering Cancer Center; UCSF; University of California San Francisco; University of California San Francisco; University of California San Francisco; University of California San Francisco; UCSF; University of California Davis; The Jackson Laboratory for Genomic Medicine; University of California San Francisco; University of California San Francisco; University of California San Francisco; UCSF; UCSF; University of California San Francisco; University of California San Francisco; University of California, San Francisco; THE JACKSON LABORATORY FOR GENOMIC MEDICINE; University of California Davis; UC Davis School of Veterinary Medicine; University of California, San Francisco

## Abstract

Meningiomas are the most common primary intracranial tumors and the only brain tumors that are more common in females compared with males1. Progestin hormonal therapies increase the risk of meningioma, and progestin-induced or pregnancy-associated meningiomas can regress as serum progestogen levels normalize2. The mechanisms that underlie sex differences and progestogen signaling in meningioma are unknown. Here we show that sex hormone interaction with PGRMC1, a transmembrane progesterone binding protein, regulates the activity of RNA processing proteins FXR1 and RBM39 to drive meningioma sex differences. The genomic architecture and stem cells underlying meningiomas are conserved across vertebrate species3–5 and, using mass spectrometry-based proteomics to analyze 703 meningioma and meningeal samples, we demonstrate that meningiomas are enriched in RNA processing proteins in humans and dogs. Interactions between PGRMC1, FXR1, and RBM39 are inhibited by progestogens and stabilized by testosterone. After release from PGRMC1, FXR1 and RBM39 bind and stabilize progesterone receptor (PR) transcript to enable expression of PR protein, which induces cell cycle, membrane, and cytoskeleton remodeling genes that drive tumor growth. These findings reveal therapeutic strategies and a PR target gene biomarker that may improve outcomes for patients. More broadly, we elucidate an estrogen receptor-independent mechanism of PR expression that underlies sex differences in cancer.

## Main text

Meningiomas, which arise from the lining of the central nervous system, comprise 42.6% of primary intracranial tumors in adults^1^. Brain magnetic resonance imaging and autopsy studies demonstrate that 2–3% of individuals will develop a meningioma in their lifetime^6–8^. Meningiomas are 2–4 times more common in females compared with males^1,9,10^, and the risk of meningioma is increased by exposure to synthetic progestins such as oral cyproterone acetate (CPA)^11–13^ and injectable medroxyprogesterone acetate (MPA)^14–20^, medications that are used for hormonal therapy and contraception in females, or for androgen suppression and treatment of prostate cancer in males.

Mechanisms linking progestogens, which include endogenous progesterone and synthetic progestins, to the development of meningioma are unknown^2,21^. Genomic studies show that progestin-induced meningiomas are enriched in DNA mutations that are associated with favorable clinical outcomes^22,23^, which suggests that progestogens may drive the growth of otherwise indolent meningioma cells. Recent investigations of DNA mutations^10,24–26^, copy number alterations (CNAs)^27–29^, DNA methylation and chromatin profiles^30–37^, bulk and single-cell gene expression^3,38–42^, and integrated models combining genomic and histological features^43–45^ have transformed understanding of meningioma biology. These studies have revealed stem cells, biological drivers, and molecular groups and subgroups of meningiomas that can evolve within individual patients^3,46–48^. Despite these scientific advances that have informed guidelines for tumor classification^49,50^ and the design of clinical trials for patients^51,52^, mechanisms driving sex differences in the incidence or growth of meningioma are unknown.

In most tissues, such as the female reproductive tract, breast, and breast cancer, estrogen receptor (ER) is required for expression of progesterone receptor (PR)^53^. Immunohistochemical (IHC) studies show that most meningiomas express PR but lack ER^54–56^, except in rare cases of ER expression in the tumor microenvironment. Cultured meningioma cells lose expression of PR *in vitro*^57^, and a randomized clinical trial showed that PR inhibition does not improve survival or attenuate tumor growth in patients with recurrent meningioma^58^. Thus, PR expression is a hallmark of meningioma in females and males, but it is unknown how PR is expressed and it is unclear if PR is a driver gene or merely a marker gene in meningioma. Nevertheless, meningiomas that are diagnosed during pregnancy or progestin therapy can regress postpartum or after progestin cessation^59–69^, which again supports the hypothesis that progestogens drive meningioma growth. Sex differences are important determinants of cancer biology^70–73^, including in brain tumors^74–79^, but genomic studies have failed to explain how meningiomas are more common in females compared with males or the role that sex hormones and PR may play in meningioma biology.

Here we develop a proteogenomic atlas (https://raleighlab.shinyapps.io/meningioma-proteogenomic-atlas/) and use mechanistic, functional, genetic, and pharmacologic approaches to study sex differences in meningioma. We find that meningiomas in humans and dogs are enriched in myriad RNA processing proteins. Like sex differences, RNA processing is an important determinant of cancer biology^80,81^. Using CRISPR interference (CRISPRi) screens, machine learning, cross-linking immunoprecipitation (CLIP) sequencing, long-read RNA sequencing, and RNA stability assays, we show that RNA processing proteins FXR1 and RBM39 drive meningioma growth in response to progestogens by binding and stabilizing progesterone receptor transcript (*PGR*) to enable expression of progesterone receptor protein (PR). Immunoprecipitation mass spectrometry (IP-MS) and structure-function studies show progestogens release FXR1 and RBM39 from PGRMC1, a poorly understood progesterone binding protein, to stabilize *PGR* and drive expression of PR. Testosterone and the small molecule AG205 interact with the binding pocket on PGRMC1 to stabilize interactions with RNA processing proteins, which destabilizes *PGR* and decreases expression of PR. We find that progestogens signal through this pathway to drive the growth of meningioma in female mice and in male mice after castration, and that testosterone, AG205, and RBM39 degradation with the small molecule indisulam block the growth of meningioma. Using chromatin immunoprecipitation (ChIP) sequencing integrated with bulk and spatial transcriptomic sequencing and proteomics, we define a 58 gene biomarker of PR activity in meningioma. Together, these studies elucidate a comprehensive proteogenomic mechanism that drives sex differences in meningioma, revealing signaling events and target genes that may enable precision medicine for patients.

### Meningiomas are enriched in RNA processing proteins

**Figure 1:**
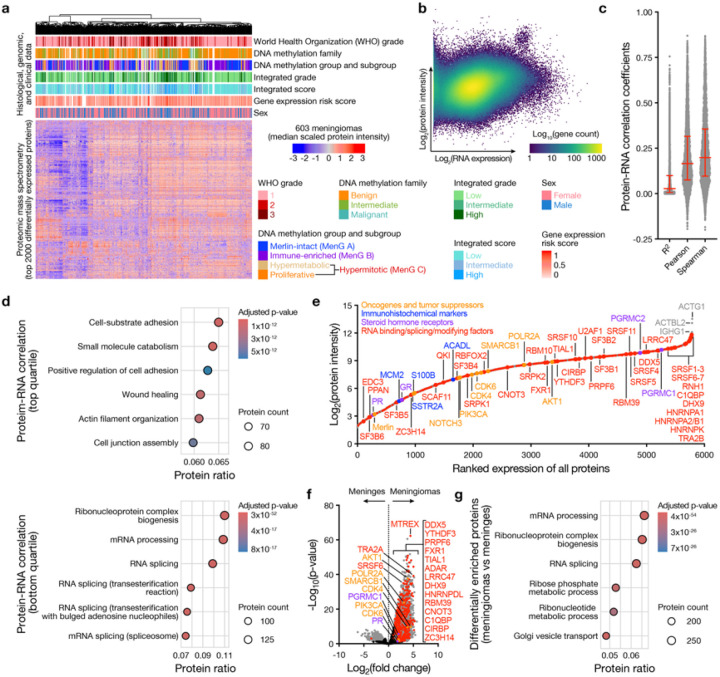
Meningiomas are enriched in RNA processing proteins. **a**, Heatmap showing unsupervised hierarchical clustering of the top 2000 differentially expressed proteins from mass spectrometry-based proteomics of 603 meningiomas with matched DNA methylation, gene expression, copy number alteration, and histological data. **b**, Scatter plot showing expression of 1,038,635 gene-by-gene protein-RNA pairs from 446 meningiomas with matched mass spectrometry-based proteomic and RNA sequencing data. **c**, Correlation coefficients for paired protein-RNA expression data. Lines represent means and error bars represent the standard error of the means. **d**, Ontology analyses of protein-RNA pairs with the highest (top) or lowest (bottom) Spearman correlation coefficients. **e**, Ranked expression of 5,788 meningioma proteins. **f**, Volcano plot showing differentially expressed proteins from mass spectrometry-based proteomics of meningiomas versus meningeal samples (n=6). As in **e**, RNA processing proteins are shown in red, oncogenes and tumor suppressors are shown in orange, and steroid hormone receptors are shown in purple. **g**, Ontology analysis of differentially enriched proteins in meningiomas versus meningeal samples.

To develop a proteogenomic atlas of meningioma, we performed mass spectrometry-based proteomics to measure protein abundances in 603 human meningioma samples with corresponding CNA, DNA methylation, short-read RNA sequencing, targeted gene expression, and histological data, from patients with demographic and clinical outcomes data^28,30,36,39,40,47^. This approach identified 6,472 proteins with ≥2 peptides per protein that were expressed in ≥25% of samples with minimal batch effects (Extended Data Fig. 1a–d). Meningioma DNA methylation profiling identifies molecular families^31^, groups^30,37,38^, and subgroups^36,45^ of tumors that can be combined with CNAs and histological data into an integrated score^44^. Alternatively, CNAs can be combined with meningioma histological data into an integrated grade^43^, and gene expression profiling refines meningioma risk stratification to identify patients who benefit from postoperative radiotherapy^40,41^. In contrast to these genomic approaches for meningioma classification, unsupervised hierarchical clustering, continuous distribution functions, silhouette plots, and *K*-means consensus clustering of differentially expressed proteins failed to reveal robust molecular groups of meningioma (Extended Data Fig. 1e–i, Supplementary Table 1). Moreover, unsupervised analysis of meningioma proteomic data did not correlate with World Health Organization (WHO) histological grade, or molecular groups of tumors (Fig. 1a). In support of these findings, gene-by-gene protein-RNA correlation in meningioma from proteomic analysis compared with RNA sequencing of the same samples was extraordinarily low, even in comparison to other cancers^82,83^, with mean R^2^, Pearson, and Spearman correlation coefficients of 0.08, 0.21, and 0.24, respectively (Fig. 1b, c). Thus, proteomic analysis sheds light on aspects of meningioma biology that may not be appreciated from genomic studies.

Ontology analyses showed that genes with high protein-RNA correlation were involved in cell membrane and cytoskeleton remodeling, and that genes with low protein-RNA correlation were involved in RNA processing (Fig. 1d). RNA processing proteins were also the most differentially expressed proteins across the proteogenomic atlas (Extended Data Fig. 1j) and ranked expression of meningioma proteins revealed enrichment of RNA binding, splicing, and modifying factors that outnumbered traditional oncogenes and tumor suppressors (Fig. 1e, Supplementary Table 2). Steroid hormone receptors were also identified by proteomics, including PR and glucocorticoid receptor (GR), which regulates meningioma apoptosis^30^, but not ER or androgen receptor (AR) (Fig. 1e, Supplementary Table 2). Differential expression analysis of proteomic data demonstrated enrichment of RNA processing proteins in human meningiomas compared with human meningeal samples (Fig. 1f, g, Supplementary Table 3). The R^2^ correlation coefficient for protein-RNA expression in the meninges was 0.51.

Meningiomas are one of the most common primary intracranial tumors in domestic animals^84^, and the molecular architecture and stem cells underlying meningioma are conserved between humans and dogs^3–5^. To validate proteomic findings from human meningiomas, we performed mass spectrometry-based proteomics on 94 dog meningioma and meningeal samples, which identified 6,694 proteins with ≥2 peptides per protein that were expressed in ≥25% of samples with minimal batch effects (Extended Data Fig. 2a–d). Unsupervised hierarchical clustering of proteomic data failed to identify robust molecular groups of dog meningiomas, and ontology analysis showed that the most differentially expressed proteins were involved in RNA processing, as in humans (Extended Data Fig. 2e, f, Supplementary Table 4). Gene-by-gene protein-RNA correlation in dog meningiomas from proteomic analysis compared with RNA sequencing of the same samples (n=20)^4^ was low, with mean R^2^, Pearson, and Spearman correlation coefficients of 0.06, 0.03, and 0.06, respectively. Ranked expression of dog meningioma proteins demonstrated enrichment of RNA binding, splicing, and modifying factors (Supplementary Table 5), and differential expression analysis of proteomic data revealed enrichment of RNA processing proteins in dog meningiomas compared with dog meningeal samples (Extended Data Fig. 2g, h, Supplementary Table 6). These findings demonstrate that RNA processing proteins are a conserved proteomic hallmark of meningioma that are expressed across species and molecular and histological groups of tumors.

### RNA processing proteins underlie meningioma growth and progesterone receptor expression

**Figure 2:**
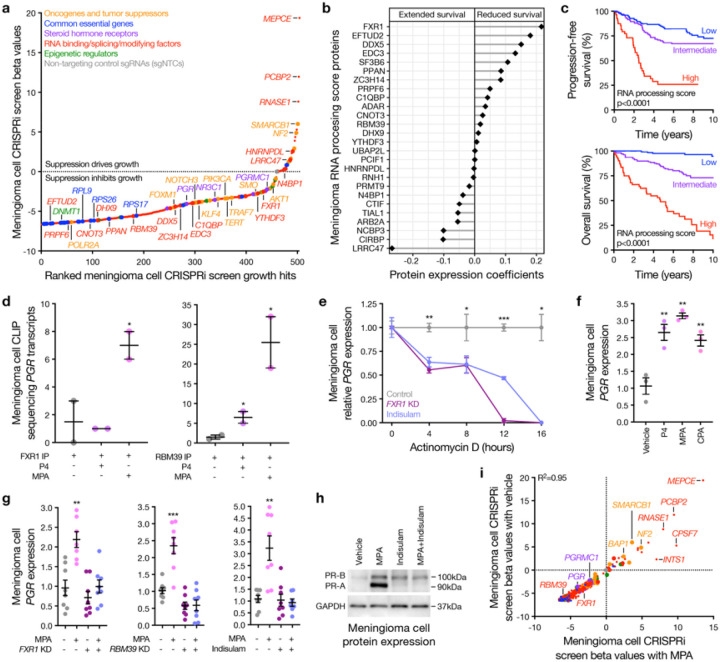
RNA processing proteins underlie meningioma growth and progesterone receptor expression. **a**, Ranked beta values from CRISPRi screening of RNA binding/splicing/modifying factors, common essential genes, and select steroid hormone receptors, epigenetic regulators, and oncogenes and tumor suppressors from human meningiomas in M10G meningioma cells stably expressing CRISPRi machinery (dCas9-Zim3). RNA binding/splicing/modifying factors included in the machine learning RNA processing score in human tumors, defined in **b** and **c**, are shown by large red circles. **b**, Development of a meningioma RNA processing risk score using LASSO regression of mass spectrometry-based proteomic data (n=425) to predict overall survival. **c**, Kaplan-Meier curves showing clinical outcomes across RNA processing score risk strata using meningioma mass spectrometry-based proteomic data (n=425). Log-rank tests. **d**, Progesterone receptor transcript (*PGR*) recovery after CLIP sequencing and treatment of M10G cells with progesterone (P4) 1μM, medroxyprogesterone acetate (MPA) 1μM, or vehicle control for 4hrs and immunoprecipitation (IP) of FLAG-tagged FXR1 or RBM39. **e**, QPCR for *PGR* expression over time in M13C meningioma cells treated with actinomycin D 5μM for the time shown ± *FXR1* siRNA knockdown (KD) or treatment with indisulam 20μM for 48hrs to degrade RBM39. Data at each timepoint are normalized to *PGR* transcript expression in control cells with actinomycin D treatment but no other genetic or pharmacologic perturbations. **f**, QPCR for *PGR* expression in CH157-MN meningioma cells treated with P4, MPA, cyproterone acetate (CPA) 1μM, or vehicle control for 7 days. **g**, QPCR for *PGR*transcript expression in CH157-MN cells after *FXR1* or *RBM39* KD or treatment with indisulam and/or MPA for 48hrs. **h**, Immunoblot for progesterone receptor protein (PR) expression in M10G cells treated with MPA and/or indisulam versus vehicle control for 14 days. **i**, Correlation of CRISPRi screen beta values from M10G cells stably expressing dCas9-Zim3 ± MPA treatment. Circle size and color defined in **a**. Lines represent means and error bars represent the standard error of the means. Student’s t-tests, *p≤0.05, **p≤0.01, ***p≤0.0001.

To determine if RNA processing proteins contribute to meningioma cell growth, we developed a CRISPRi screening library (n=2990 sgRNAs) to target (1) the 447 RNA binding, splicing, and modifying factors identified by meningioma proteomics, (2) 29 meningioma-specific tumor suppressor genes or oncogenes from genomic studies, including steroid hormone receptors and epigenetic regulators, (3) 30 common essential genes, and (4) 250 non-targeted sgRNAs (sgNTCs) (Supplementary Table 7). CRISPRi screening in patient-derived meningioma cells demonstrated that most RNA processing genes (n=416, 93.1%), oncogenes, steroid hormone receptors, and epigenetic regulators promoted meningioma cell growth, and that tumor suppressors, such as *NF2* and *SMARCB1* which are often inactivated in meningioma^10^, and a minority of RNA processing genes (n=31, 6.9%) inhibited meningioma cell growth (Fig. 2a, Supplementary Table 8).

To validate these findings in patients, we used least absolute shrinkage and selection operator (LASSO) machine learning regression with 10-fold cross-validation and overall survival as the target endpoint to develop a meningioma RNA processing risk score. An optimized set of 26 RNA processing proteins was identified and tested in separate discovery (Fig. 2b, c) and validation (Extended Data Fig. 3a) cohorts comprised of clinical and proteomic data from 425 and 176 patients, respectively (Supplementary Table 9). A highly discriminatory set of linearly rescaled risk scores between 0 and 1 that were associated with overall and progression free survival were identified for the 26 RNA processing proteins in the risk score, and a nested rank statistic was used for delineation of discrete RNA processing risk groups (Fig. 2c, Extended Data Fig. 3a). Phenotypic concordance between the 26 RNA processing proteins in patients and CRISPRi screen results in meningioma cells was 80.8%. Among concordant RNA processing proteins, FXR1 and RBM39 promoted meningioma cell growth (Fig. 2a) and were associated with reduced survival in patients (Fig. 2b). FXR1, an RNA binding protein that regulates RNA stability and translation^85^, was the strongest driver of reduced survival in patients, and RBM39, an RNA binding protein that regulates RNA splicing and transcription, can be selectively targeted for degradation with indisulam^86^.

CLIP sequencing was used to define RNA transcript interactions with FLAG-tagged FXR1 or RBM39 in patient-derived meningioma cells, which revealed a diversity of 5’ UTR, 3’ UTR, exon, and intron binding events in transcripts of genes that were involved in cell cycle progression, cellular metabolism, and intracellular transport (Extended Data Fig. 3b–d, Supplementary Table 10). CLIP sequencing further demonstrated that FXR1 and RBM39 each bound to *PGR* in meningioma cells, and that interactions between RNA processing proteins and *PGR* were increased upon treatment of meningioma cells with progesterone (P4) or MPA (Fig. 2d).

RNA splicing is dysregulated in cancer^80^, and short-read RNA sequencing demonstrates that alternative RNA splicing events are differentially enriched in molecular groups of meningioma^87^. To our knowledge, long-read RNA sequencing, which provides comprehensive information about alternative RNA splicing (Extended Data Fig. 4a), has not been reported for meningioma. Thus, to determine how RNA processing proteins might regulate *PGR* in meningioma, we performed long-read RNA sequencing on 84 human meningioma samples with histological and genomic data from patients with available demographic and clinical data (Supplementary Table 11). Unsupervised hierarchical clustering, differential expression, and ontology analyses of alternative RNA splicing events revealed that transcripts of genes involved in apoptosis or cellular metabolism were alternatively spliced in meningioma (Extended Data Fig. 4b, c, Supplementary Table 12). Most RNA splicing events in meningioma targeted alternative first or last exons across demographic, clinical, histological, and molecular groups (Extended Data Fig. 4d). Long-read RNA sequencing also showed predominant expression of the same *PGR* transcript variants across demographic, clinical, histological, and molecular groups of meningiomas (Extended Data Fig. 5a, b, Supplementary Table 13), suggesting that FXR1 and RBM39 do not regulate alternative splicing of *PGR* in meningioma.

The transcription factor FOXM1 drives meningioma cell growth^30,39^, and the RNA binding protein IGF2BP1 stabilizes expression of FOXM1 target gene transcripts in meningioma cells^88^. To determine if FXR1 and RBM39 similarly stabilize *PGR*, actinomycin D was used to block new transcript expression after siRNA suppression of RNA processing proteins (Extended Data Fig. 5c) or degradation of RBM39 with indisulam (Extended Data Fig. 5d). Genetic and pharmacologic inhibition of FXR1 or RBM39 accelerated *PGR* degradation in patient-derived meningioma cells (Fig. 2e). In ER+/PR+ breast cancer cells, genetic and pharmacologic inhibition of FXR1 or RBM39 transiently influenced *PGR* expression and degradation (Extended Data Fig. 5e). In support of these findings, progestogen treatment of meningioma cells expressing FXR1 and RBM39 increased the expression of *PGR* (Fig. 2f) and PR (Extended Data Fig. 5f), but genetic or pharmacologic inhibition of RNA processing proteins blocked *PGR* and PR expression (Fig. 2g, h). Repeat CRISPRi screening in patient-derived meningioma cells during treatment with MPA showed high correlation with growth screen results (R^2^ correlation coefficient 0.95) and demonstrated that suppression of *PGR* inhibited the growth of meningioma cells (Fig. 2a, i, Supplementary Table 8). These findings reveal that RNA processing proteins FXR1 and RBM39 regulate *PGR* stability and expression to drive the growth of meningioma cells in response to progestogens.

### Progestogens block PGRMC1 interaction with RNA processing proteins to enable expression of progesterone receptor in meningioma

**Figure 3:**
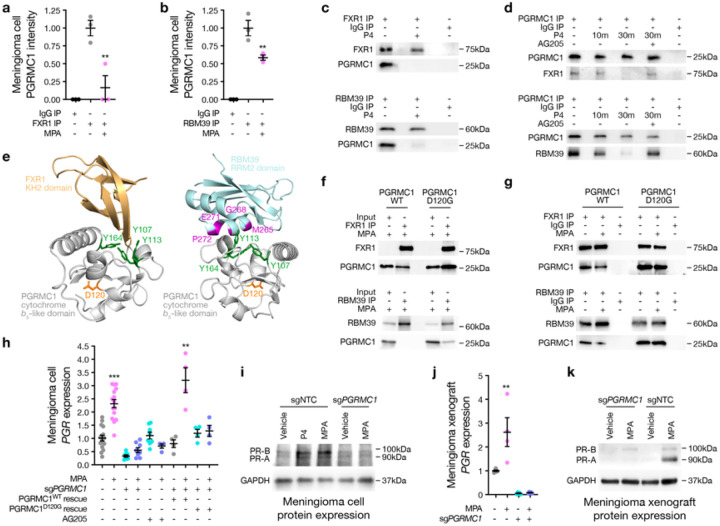
Progestogens block PGRMC1 interaction with RNA processing proteins to enable expression of progesterone receptor in meningioma. **a**, Immunoprecipitation mass spectrometry (IP-MS) for FXR1 protein interactions in CH157-MN meningioma cells treated with medroxyprogesterone acetate (MPA) 1μM versus vehicle control for 30min. **b**, IP-MS for RBM39 protein interactions as in **a**. **c**, FXR1 or RBM39 immunoprecipitation (IP) for co-IP immunoblots of PGRMC1 from CH157-MN cells treated with progesterone (P4) 1μM versus vehicle control for 30 min. **d**, PGRMC1 IP for co-IP immunoblots of FXR1 or RBM39 from CH157-MN cells treated with P4 ± AG205 10μM for 30min. **e**, Structural modeling of full-length proteins showing the cytochrome *b*_*5*_-like domain of PGRMC1 (grey residues 72–171), which binds to progesterone (green and orange residues), interacts with the KH2 domain of FXR1 (gold residues 285–314, left) and the RRM2 domain of RBM39 (aqua residues 250–328, right). Indisulam binding residues on RBM39 are shown in purple. **f**, FXR1 or RBM39 IP for co-IP immunoblots of exogenous PGRMC1 from CH157-MN cells stably expressing CRISPRi machinery (dCas9-KRAB), sgRNAs targeting endogenous *PGRMC1*, rescue of either wildtype (WT) or D120G-mutant PGRMC1, and treatment with MPA showing more PGRMC1^D120G^ than PGRMC1^WT^ in complex with RNA processing proteins. **g**, FXR1 or RBM39 IP for co-IP immunoblots of exogenous PGRMC1 from cells in **f** ± treatment with MPA 1μM for 30min validating that PGRMC1^D120G^ does not dissociate from RNA processing proteins in response to MPA while PGRMC1^WT^ does. **h**, QPCR for progesterone receptor transcript (*PGR*) expression in CH157-MN cells with CRISPRi suppression of *PGRMC1* versus expression of non-targeted control sgRNAs (sgNTC), rescue of WT versus D120G PGRMC1, or treatment with AG205 10μM ± MPA 1μM for 48hrs. **i**, Immunoblot for progesterone receptor protein (PR) expression in CH157-MN cells with CRISPRi suppression of *PGRMC1* ± treatment with P4 1μM or MPA for 14 days. **j**, QPCR for *PGR* expression in IOMM-Lee meningioma xenografts grown in female *Nu/Nu* mice ± CRISPRi suppression of *PGRMC1* in meningioma cells stably expressing dCas9-Zim3 ± treatment of mice with MPA 10mg/kg daily via IP injection versus vehicle control for 30 days. **k**, Immunoblot for PR expression in IOMM-Lee xenografts grown in female *Nu/Nu* mice with CRISPRi suppression of *PGRMC1* in meningioma cells ± treatment of mice with MPA versus vehicle control for 25 days. Lines represent means and error bars represent the standard error of the means. Student’s t-tests, **p≤0.01, ***p≤0.0001.

Meningioma proteomic analysis showed that FXR1 and RBM39 were expressed across all demographic, clinical, histological, and molecular groups of tumors, and that the expression of these RNA processing proteins did not correlate with *PGR* or PR expression in patients (Extended Data Fig. 6a, b). To test the hypothesis that differentially expressed upstream factors may regulate FXR1 and RBM39 activity in response to progestogens, IP-MS was used to define endogenous FXR1 and RBM39 protein interactions in meningioma cells (Extended Data Fig. 7a, b, Supplementary Table 14). Progesterone receptor membrane component 1 (PGRMC1), a progesterone binding protein that is expressed across multiple cancers^89,90^, was the only steroid hormone receptor that was identified in FXR1 and RBM39 IP-MS samples, and IP-MS interactions between RNA processing proteins and PGRMC1 decreased when meningioma cells were treated with MPA before FXR1 or RBM39 immunoprecipitation (Fig. 3a, b). These findings were validated using IP-MS for endogenous PGRMC1 in the same (Supplementary Table 15) and other (Extended Data Fig. 8a) meningioma cell lines, which demonstrated that FXR1 and RBM39 were the only proteins that consistently decreased in intensity in PGRMC1 IP-MS samples after MPA treatment of meningioma cells. Secondary validation was performed using reciprocal immunoprecipitation immunoblots for endogenous FXR1, RBM39, and PGRMC1 from meningioma cells, which showed that progesterone and MPA blocked RNA processing protein interaction with PGRMC1 (Fig. 3c, Extended Data Fig. 8b), and that AG205, a small molecule that inhibits progesterone binding to PGRMC1^91^, stabilized PGRMC1 interactions with RNA processing proteins (Fig. 3d). Proteomic analysis demonstrated that PGRMC1 expression correlated with *PGR* and PR expression in meningioma, unlike FXR1 and RBM39 (Extended Data Fig. 6b).

Structural modeling with AlphaFold3^92^ suggested that the cytochrome *b*_*5*_-like domain of PGRMC1, which binds to progesterone^93^, interacts with the KH2 domain of FXR1, which regulates RNA binding and stability, and the RRM2 domain of RBM39, which regulates RNA binding and interaction with indisulam (Fig. 3e). Structural modeling and ligand affinity prediction with Boltz2^94^ and Chai-1^95^ validated these interactions, and showed polar and aromatic interactions between the cytochrome *b*_*5*_-like domain of PGRMC1 and progestins, such as CPA and MPA, that increase the risk of meningioma from epidemiological studies^11–20,96^ (Extended Data Fig. 8c, Supplementary Table 16). Structural modeling also suggested that AG205 stabilizes interactions between the cytochrome *b*_*5*_-like domain of PGRMC1 and the KH2 domain of FXR1 or the RRM2 domain of RBM39 (Extended Data Fig. 8d). To test these models, endogenous *PGRMC1* was suppressed in meningioma cells using CRISPRi (Extended Data Fig. 8e), and constructs encoding either wildtype (WT) PGRMC1 or PGRMC1 with mutation of aspartic acid 120 to glycine (D120G) in the cytochrome *b*_*5*_-like domain, which blocks progesterone binding^93^, were expressed. Immunoprecipitation and immunoblots demonstrated that MPA blocked interactions between PGRMC1^WT^ and RNA processing proteins, but that MPA could not decouple RNA processing proteins from PGRMC1^D120G^ in meningioma cells (Fig. 3f, g).

Analysis of meningioma H3K27Ac ChIP-sequencing data (n=33)^32^ showed open chromatin at genomic loci for *PGRMC1*, *FXR1, RBM39, PGR*, *NF2,* and *FOXM1,* and closed chromatin at genomic loci for estrogen receptor (*ESR1*) and *AR* (Extended Data Fig. 9a). Proteomic analysis demonstrated enrichment of PGRMC1 in meningiomas from women, newly diagnosed meningiomas, and meningiomas from favorable histological and molecular groups, following the same patterns as PR (Extended Data Fig. 6a, 9b). PGRMC1 was the most expressed steroid hormone receptor in meningioma (Fig. 1e, Supplementary Table 2) and PGRMC1 was differentially enriched in meningiomas compared with meningeal samples (Fig. 1f). Immunofluorescence microscopy revealed that PGRMC1, a single-pass transmembrane protein (Extended Data Fig. 8c), was expressed in intracellular meningioma cell membranes and was co-expressed with PR in human tumor sections (Extended Data Fig. 9c, d). CRISPRi screening showed that *PGRMC1* promoted meningioma cell growth, like *PGR* (Fig. 2a, i), and targeted CRISPRi suppression of *PGRMC1* attenuated meningioma cell growth in response to MPA compared with meningioma cells expressing sgNTC (Extended Data Fig. 9e).

PGRMC1 appears to signal through EGFR and AKT in breast cancer cells^97^, but there were no changes in EGFR or AKT phosphorylation with MPA treatment of meningioma cells with or without CRISPRi suppression of *PGRMC1* (Extended Data Fig. 9f). Instead, CRISPRi suppression or pharmacological inhibition of PGRMC1 with AG205 blocked expression of *PGR* and PR in meningioma cells treated with MPA compared with vehicle control (Fig. 3h, i), and *PGR* expression in response to MPA was restored by rescue of PGRMC1^WT^ but not PGRMC1^D120G^ (Fig. 3h). CRISPRi suppression of *PGRMC1* also blocked the expression of *PGR* and PR in meningioma xenografts implanted in female mice that were treated with MPA compared with vehicle control (Fig. 3j, k). Proteomic analysis showed that meningiomas also express PGRMC2 (Fig. 1e, Supplementary Table 2), a heme chaperone with no transmembrane domain, a different cytochrome domain than PGRMC1, and no known hormone interactions^98^. CRISPRi suppression of *PGRMC2* did not block expression of *PGR* or attenuate meningioma cell growth in response to MPA compared with vehicle control (Extended Data Fig. 9g-i).

### Sex hormones signal through PGRMC1, RNA processing proteins, and progesterone receptor to regulate the expression of cell cycle and membrane and cytoskeletal remodeling genes that drive meningioma

**Figure 4:**
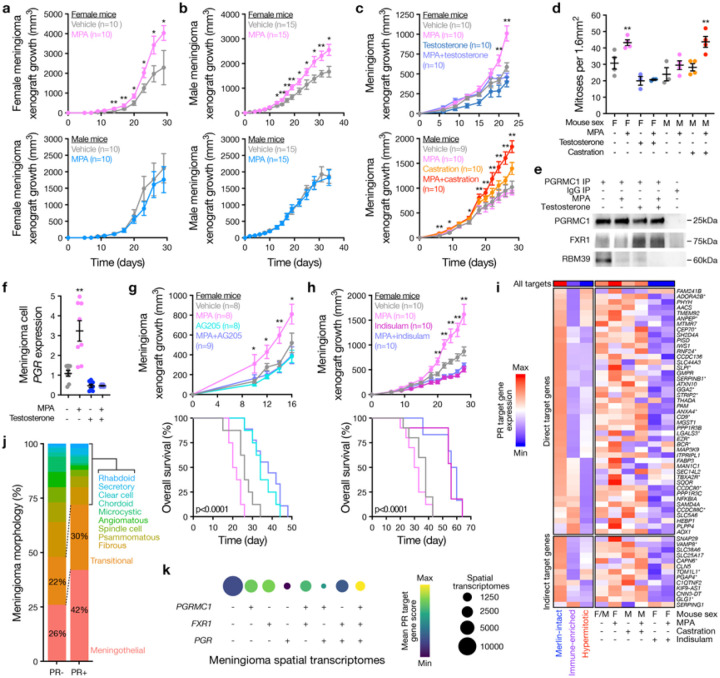
Sex hormones signal through PGRMC1, RNA processing proteins, and progesterone receptor to regulate the expression of cell cycle and membrane and cytoskeletal remodeling genes that drive meningioma. **a**, Growth of CH157-MN female meningioma xenografts in female (top) versus male (bottom) *Nu/Nu* mice treated with medroxyprogesterone acetate (MPA) 10mg/kg daily via IP injection versus vehicle control. **c**, Growth of IOMM-Lee male meningioma xenografts in female (top) versus male (bottom) *Nu/Nu* mice treated as in **a**. **c**, Growth of IOMM-Lee xenografts in female (top) versus male (bottom) *Nu/Nu* mice treated with MPA, testosterone subcutaneous pellet (5mg for 60 days) with or without MPA in female mice, MPA with or without castration of male mice, or vehicle control. **d**, Histological quantification of xenograft mitoses from **c** after 30 days of treatment. **e**, PGRMC1 immunoprecipitation (IP) for co-IP immunoblots of FXR1 or RBM39 from CH157-MN cells treated with MPA 1μM and/or testosterone 10μM versus vehicle control for 30min. **f**, QPCR for progesterone receptor transcript (*PGR*) transcript expression in CH157-MN cells after treatment with MPA and/or testosterone versus vehicle control for 48hrs. **g**, IOMM-Lee xenograft growth (top) and overall survival (bottom, Log-rank test) in female *Nu/Nu* mice treated with MPA and/or AG205 10mg/kg daily via IP injection versus vehicle control. AG205 treatment was stopped after 16 days. **h**, IOMM-Lee xenograft growth (top) and overall survival (bottom, Log-rank test) in female *Nu/Nu* mice treated with MPA and/or indisulam 25mg/kg daily via IP injection versus vehicle control. Indisulam treatment was stopped after 30 days. **i**, Heatmap showing PR target gene expression from RNA sequencing and WGCNA analysis of 200 meningiomas with matched mass spectrometry-based proteomic and DNA methylation profiling data (left) or from RNA sequencing of xenografts from **g** and **h** (right). Cell cycle and remodeling genes are marked with asterisks. **j**, Stacked bar plots showing the morphologic distribution of 276 meningiomas with available histological and mass spectrometry-based proteomic data (Chi-squared test, p=0.0338). **k**, PR target gene expression across 28,235 spatial transcriptomes with or without *PGRMC1, FXR1,* and/or *PGR* expression from 8 meningiomas. Lines represent means and error bars represent the standard error of the means. Student’s t-tests, *p≤0.05, **p≤0.01.

During the course of *in vitro* experimentation, we observed that meningioma cells grew slower in male compared with female bovine serum (Extended Data Fig. 10a) and that progesterone or progestin treatment increased the growth of meningioma cells (Extended Data Fig. 10b). To study sex differences in meningioma *in vivo*, meningioma cells from either male or female patients were implanted in either male or female mice that were treated with MPA or vehicle control. There was no difference in meningioma xenograft growth according to patient sex in either male or female mice treated with vehicle control (Extended Data Fig. 10c, d), and MPA had no impact on meningioma xenograft growth in male mice, but MPA accelerated the growth of meningioma xenografts from either male or female patients in female mice (Fig. 4a, b). CRISPRi suppression of *PGRMC1* or *PGR* blocked meningioma xenograft growth in response to MPA (Extended Data Fig. 10e-g). Rescue expression of PGRMC1^WT^ but not PGRMC1^D120G^, or over-expression of PGR, restored meningioma cell growth in response to MPA after CRISPRi suppression of *PGRMC1* (Extended Data Fig. 10h, i).

These data show that progestins signal through PGRMC1 and PR to drive the growth of meningioma by a mechanism that is conserved in tumor cells from male and female patients and is either inhibited in males or activated in females. Proteomic analysis demonstrated that PGRMC1 and PR, but not ER, were expressed in meningiomas from males and females (Extended Data Fig. 6a, 9b, Supplementary Table 2), and estrogen treatment did not accelerate meningioma xenograft growth in male or female mice or in male mice with concurrent MPA treatment compared with vehicle control (Extended Data Fig. 10j). Thus, to test the hypothesis that progestin signaling in meningioma may be inhibited in males, MPA treatments were repeated with or without testosterone treatment of female mice or castration for depletion of testosterone in male mice. Testosterone inhibited meningioma xenograft growth in response to MPA in female mice, and castration accelerated meningioma xenograft growth and disinhibited response to MPA in male mice (Fig. 4c). Histological and IHC analyses of meningioma xenografts showed that MPA increased meningioma cell mitoses in female mice, and that castration and MPA increased meningioma cell mitoses in male mice, all without affecting meningioma cell apoptosis or macrophage number in the tumor microenvironment (Fig. 4d, Extended Data Fig. 10k). In support of these findings, RNA sequencing deconvolution demonstrated no difference in the immune microenvironment between PR+ and PR− meningiomas from male or female patients (Extended Data Fig. 10l).

Immunoprecipitation for PGRMC1 and immunoblotting for RNA processing proteins from meningioma cells showed that testosterone stabilized PGRMC1 interactions with FXR1, but not RBM39, with or without MPA (Fig. 4e). Structural modeling and ligand affinity prediction with calculation of backbone distances from the top 5 highest confidence models suggested that the cytochrome *b*_*5*_-like domain of PGRMC1 also interacts with testosterone, and that testosterone stabilizes interaction between PGRMC1 and the Tud1 and Tud2 domains of FXR1, which regulate protein-protein interactions (Extended Data Fig. 10m-o, Supplementarty Table 16). In support of these models, testosterone treatment attenuated *PGR* expression and meningioma cell growth in response to MPA compared with vehicle control (Fig. 4f, Extended Data Fig. 10p), and castration disinhibited PR expression in meningioma xenografts in male mice treated with MPA (Extended Data Fig. 10q). Review of the University of California San Francisco electronic medical record for all patients with a diagnosis of meningioma and serum testosterone measurement within 1 year of brain imaging prior to any meningioma treatment (n=123) demonstrated that males with normal to high testosterone had smaller meningiomas compared with females or males with low testosterone, including males treated with androgen deprivation therapy for prostate cancer (Extended Data Fig. 11a-c). Like testosterone, AG205 and indisulam blocked meningioma xenograft growth, prolonged survival, and inhibited meningioma PR expression *in vivo* (Fig. 4g, h, Extended Data Fig. 10q).

PR drives breast cancer cell proliferation through (1) phosphorylation by c-Src at serine 345 (S345) to activate the MAPK pathway^99,100^ and (2) dimerization and translocation to the nucleus to activate gene expression^101–104^. Treatment of meningioma cells with MPA induced PR^S345^ phosphorylation, MAPK activation that was inhibited by site directed mutagenesis to block PR interaction with c-Src^99,100^, and expression of cyclin D1 (Extended Data Fig. 12a-c). Subcellular fractionation of meningioma cells showed enrichment of PR in the nucleus after treatment with MPA compared with vehicle control (Extended Data Fig. 12d, e).

To identify PR target genes in meningioma, ChIP and RNA sequencing were performed after expression of PR-A and PR-B in meningioma cells. PR-A and PR-B, which were both expressed in human meningiomas (Extended Data Fig. 12f) and meningioma cells (Fig. 2h, 3i, 3k, Extended Data 5f, 10p, 12d, 12e), are transcriptionally distinct isoforms whose relative expression and activity are dysregulated in cancer^105–107^. PR-B promotes proliferative gene expression and PR-A has less defined roles in regulating proliferation and steroid hormone receptor expression. To define PR target genes in meningioma, PR-A and PR-B were expressed in meningioma cells and PR ChIP sequencing was performed after treatment with MPA (Extended Data Fig. 13a, b, Supplementary Table 17). Genes with peaks from PR ChIP sequencing were intersected with differentially expressed genes from RNA sequencing of meningioma cells with expression or CRISPRi suppression of *PGR* and treatment with MPA compared with vehicle control (Extended Data Fig. 13c, Supplementary Table 18). Ontology analysis showed that direct PR target genes with peaks from ChIP sequencing and differential enrichment after MPA treatment from RNA sequencing were involved in cell membrane and cytoskeleton remodeling, including actin dynamics and cell junction assembly (Extended Data Fig. 13d). In support of these findings, MPA treatment and *PGR* expression in meningioma cells increased actin filament length and adherens junction abundance that was blocked by CRISPRi suppression of *PGR* (Extended Data Fig. 13e-g).

To translate these findings and develop a biomarker to track progesterone signaling and response to therapy in patients, weighted gene correlation network analysis (WGCNA) of RNA sequencing data from human meningioma samples with matched proteomic, genomic, demographic, and histological data (Fig. 4i, Extended Data Fig. 14a-c) was integrated with RNA sequencing data from meningioma xenografts (Extended Data Fig. 14d, e, Supplementary Table 19) and ChIP and RNA sequencing data from meningioma cells (Extended Data Fig. 13a-d, 14e, Supplementary Table 17, 18). PR target genes in human meningiomas were identified using hierarchical clustering of RNA sequencing data, which revealed two WGCNA modules that were comprised of 58 genes which were enriched in (1) meningiomas with high PGRMC1, *PGR*, and PR expression, (2) meningioma xenografts from female mice after treatment with MPA, or meningioma xenografts from male mice after castration with or without treatment with MPA, and (3) meningioma cells expressing PR after treatment with MPA (Fig. 4i, Extended Data Fig. 14a, e). WGCNA modules were distinguished by expression of direct or indirect PR target genes, which were defined based on the presence or absence of peaks from PR ChIP sequencing of meningioma cells, respectively (Extended Data Fig. 13b-d, Supplementary Table 17). The 58 genes comprising the biomarker of PR activity in meningioma were suppressed in meningeal samples (Extended Data Fig. 14b), showed high protein-protein correlation with PGRMC1, FXR1, RBM39, and PR in meningioma (Extended Data Fig. 14c), and were suppressed by indisulam treatment of xenografts in mice (Fig. 4i, Extended Data Fig. 14e). PR target genes were involved in cell cycle, membrane, and cytoskeleton remodeling and were enriched in meningiomas from favorable histological and molecular groups (Fig. 4i, Extended Data Fig. 14a), like PGRMC1, *PGR*, and PR (Extended Data Fig. 6a, 9b).

The WHO recognizes 15 morphological variants of meningioma histology^49^, and histological analysis of meningiomas with proteomic data showed that PR+ meningiomas were more likely to display transitional or meningothelial morphology than PR− meningiomas (Fig. 4j). These morphological variants are characterized by sheets of uniform, polygonal cells and fibroblastic features, which supports the finding that PR target genes are involved in cell membrane and cytoskeletal remodeling. IHC staining for PR is often heterogeneous within individual meningiomas^54–56^. Thus, to further validate the PR target gene biomarker, spatial transcriptomic sequencing was performed on 28,235 transcriptomes from 8 meningiomas with heterogeneous IHC staining for PR (Extended Data Fig. 14f, Supplementary Table 20). The 58 gene biomarker was enriched in spatial transcriptomes that expressed *PGRMC1*, RNA processing proteins, and *PGR*, but was suppressed in spatial transcriptomes that lacked expression of one or more of these genes (Fig. 4k).

## Discussion

**Figure 5:**
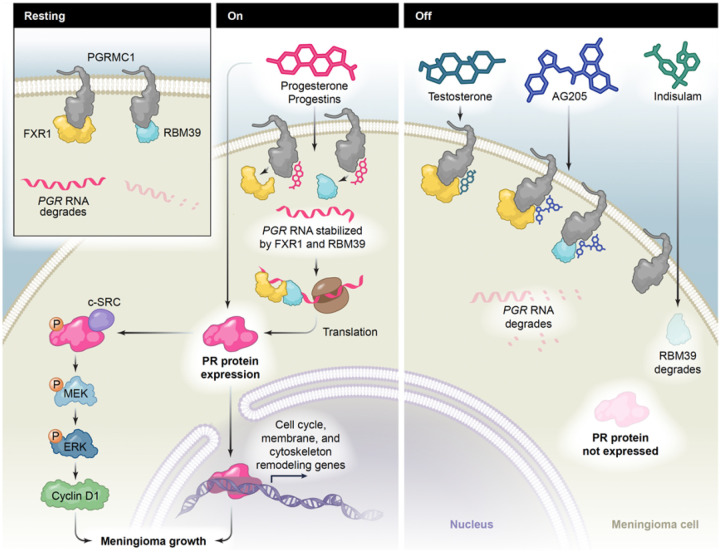
A proteogenomic RNA processing mechanism drives sex differences in meningioma. Model figure showing how sex hormones signal through PGRMC1, RNA processing proteins, and progesterone receptor to regulate the expression of progesterone receptor transcript (*PGR*), progesterone receptor protein (PR), and cell cycle, membrane, and cytoskeleton remodeling genes that drive meningioma growth. Progestogens such as progesterone and progestins activate (left), and testosterone, AG205, and indisulam inhibit (right) this pathway of ER-independent PR expression that underlies sex differences in the most common primary intracranial tumor.

Here we define mechanisms underlying sex differences in meningioma, revealing therapeutic strategies and a gene expression biomarker that may improve outcomes for patients, and an ER-independent mechanism of PR expression that is activated by progestogens and inhibited by testosterone (Fig. 5). Using mass spectrometry-based proteomics to analyze 703 meningioma and meningeal samples from humans and dogs, we demonstrate that meningiomas are enriched in RNA processing proteins. Meningioma long-read RNA sequencing further reveals enrichment of alternatively spliced transcripts that are not captured by short-read approaches. These findings shed light on limitations of previous attempts to translate the genomic classification of meningioma to IHC using supervised analysis of proteomic data from a limited number of samples without independent validation^45,108–113^. The proteogenomic atlas we develop and validate using independent, evolutionarily diverse meningiomas further reveals that PGRMC1 and PR are enriched in newly diagnosed, low grade, molecularly favorable tumors. This may explain why a clinical trial of PR inhibition in recurrent meningioma, that was performed without histological grading or molecular classification of tumors, failed to demonstrate efficacy^58^. Paradoxical activation of PR-induced transcription by the inhibitor used in this trial may have also contributed to the lack of efficacy^114^. It is possible that blocking expression of *PGR* itself with AG205, testosterone, or indisulam, which has been investigated in phase 2 clinical trials^115,116^, could improve outcomes for patients with meningiomas that are enriched in PR target genes.

This study adds to our growing understanding of meningioma biology and RNA processing in cancer^80,81^. We establish RNA processing proteins as a conserved hallmark of meningioma that are expressed across molecular and histological groups of tumors. Our findings further suggest that post-transcriptional or translational control may underly the remarkably poor protein-RNA expression correlation in meningiomas from humans and dogs. To our knowledge, RNA processing proteins have not been examined as drivers of sex differences in cancer. We show that sex hormones signal through PGRMC1 to control the activity of RNA processing proteins FXR1 and RBM39 that regulate *PGR* stability, PR expression, and gene expression programs that drive tumor growth. The activity of other RNA processing proteins in meningioma remains to be defined, but our findings from functional genomic experiments and machine learning analyses of proteomic and clinical data demonstrate that meningioma is an attractive model to study RNA biology in cancer. Some aspects of RNA processing, such as alternative splicing^87^, are enriched in different histological or molecular groups of meningioma. Our discovery that RNA processing proteins are among the most expressed proteins across all groups of meningioma suggests that future investigations of post-transcriptional or translational control will reveal additional insights.

Genetically engineered mouse models of meningioma are limited by poor penetrance and long latency^3,117^, with the exception of rare pediatric meningioma models^118^. Mutations that are enriched in progestin-associated meningiomas in humans have been reported to induce^119^ or not induce^120^ meningiomas in mice, and progestin treatment is insufficient for murine meningeal tumorigenesis^119^. Like the brain, the cellular architecture and developmental trajectory of the meninges is different between humans and mice^121–123^, and as meningiomas are not protected by the blood brain barrier^124^, the xenografts in our study provided an opportunity for interrogation of the proteogenomic mechanism we report. Investigation of potential meningioma driver mutations in non-tumor meningeal samples from females compared with males^125^, and studies of meningeal tumorigenesis using other preclinical human models, such as organoids or slice cultures, could provide further insights into functional connections between meningioma genomics and the impact of sex hormones on tumor initiation or growth.

Meningioma cells variably express *PGRMC1*, *FXR1*, *RBM39*, and *PGR* in the absence of progestogens (Extended Data Fig. 15a), and serum progesterone and testosterone levels are heterogeneous in patients with meningioma (Extended Data Fig. 15b). Thus, clinical translation of the findings from this study will require careful tumor- and patient-selection, with a likely emphasis on newly diagnosed meningiomas in patients with high serum progesterone or low serum testosterone levels. The immune system may need to be considered in future investigations, but IHC and bioinformatic deconvolution of the meningioma immune microenvironment in our study revealed no differences according to patient or mouse sex, or meningioma PR expression.

Our study has important implications for current clinical practice. Our mechanistic and functional data from meningioma cell and xenograft models provide explanations for sex differences in meningioma incidence^1^ and for how progestin-induced or pregnancy-associated meningiomas can regress as serum progestogen levels normalize^2^. Our findings further suggest that meningiomas might not be more common in females compared with males but for elevated progestogen exposure. Pregnancy is not an independent risk factor for meningioma^126^, but exposure to CPA or injectable MPA^14–20^ increases the risk of meningioma diagnosis. CPA is a progestin and antiandrogen that increases the risk of meningioma in females receiving hormonal therapy^11,13^ and in males with prostate cancer^12^, and CPA can drive the growth of meningioma during male-to-female gender affirming care^127^. Injectable MPA is used for contraception by 20–25% of sexually active females in the United States and 68–74 million females worldwide^14,18,128–130^. Meningiomas are the only brain tumors that are more common in non-Hispanic Black females compared with non-Hispanic White females^131,132^, and injectable MPA use is higher among non-Hispanic Black females^133,134^. Our finding that progestogens signal through PGRMC1 to drive meningioma expression of PR and tumor growth establishes a mechanistic framework for interpreting these epidemiological data. Moreover, our data underscore the importance of adequately counseling patients about the risk of meningioma, monitoring patients using CPA or injectable MPA for signs of meningioma, and discontinuing these medications if meningioma is diagnosed, especially if safe alternatives that are acceptable to patients are available.

Our study lays the groundwork for future investigation. We show that PGRMC1 and RNA processing proteins contribute to *PGR* expression in breast cancer cells, which elucidates a molecular mechanism that may explain rare cases of ER−/PR+ breast cancer (Extended Data Fig. 15c). Structural models showing that AG205 and testosterone stabilize PGRMC1 interactions with RNA processing proteins to block tumor growth provides a foundation for rational drug design. The AR-independent mechanism of action for testosterone that we report warrants future investigation, including in genetically engineered mouse models that decouple sex chromosomes from gonadal sex hormone production^74^. RNA proteins themselves are subjected to extensive translational control and post-translational modification^80,81^, and regulation of protein stability and dynamic complex formation were not captured by the steady-state RNA and protein measurements here but could be studied using stable isotope tracing in preclinical models or patients^135^. Meningiomas are associated with significant intratumor heterogeneity^46–48,136^, and how meningioma protein expression may change during tumor growth, spatial evolution, or response to therapy is unknown.

In summary, we have identified a proteogenomic RNA processing mechanism that drives sex differences in meningioma, the most common primary intracranial tumor. These findings elucidate mechanisms explaining longstanding epidemiological and clinical observations for one of the only brain tumors that is more common in females compared with males. The therapeutic strategies and target gene biomarker we report establish tools for precision medicine. We are hopeful that broader investigation of PGRMC1 and RNA processing will yield additional fundamental observations about cancer sex differences and will teach us which patients might benefit most from biomarker-guided targeted therapies.

## Methods

### Ethics and institutional approval

This study complied with all relevant ethical regulations and was approved by the University of California San Francisco Institutional Review Board (15–17500, 18–24633), and the University of California San Francisco Institutional Animal Care and Use Committee (AN203916). As part of routine clinical practice, all patients included in this study signed a written waiver of informed consent to contribute de-identified tissue and data for research. Tissue samples from dog meningioma patients were obtained under informed owner consent from clinical cases presenting to the University of California Davis Veterinary Medical Teaching Hospital.

### Meningioma mass spectrometry-based proteomics

Frozen meningioma samples were solubilized in 2% SDS and 100mM Tris-HCl (pH 8.0), sonicated at 40% Ampl for 15s on ice (Q700, QSonica), rotated at 4°C for 30min, and centrifuged at 15,000rcf at 4°C for 15min. Supernatant was collected, concentration, and quantified using the BCA assay (23225, Pierce), and 200mg of protein per sample was diluted into 100mL total SDS-Tris buffer. Samples were reduced in 10mM DTT (D0632, Sigma-Aldrich), alkylated with 25mM iodoacetamide (I1149, Sigma-Aldrich), and subjected to tryptic hydrolysis using the HILIC bead SP3 protocol^137^. In brief, 20μg of 50:50 hydrophilic:hydrophobic Sera-mag SP3 beads were added to each sample (45152105050250 and 65152105050250, Cytiva). The bead-protein mixture was acidified to pH ~2 with formic acid, and acetonitrile was added to a reach a final concentration of 50% (v/v). Mixtures were incubated for 10min at room temperature and then placed on a magnetic rack. The supernatant was discarded and beads were rinsed twice with 200μl of 70% ethanol and three times with 200μl acetonitrile. Rinsed beads were reconstituted in 10μL of 25mM Tryspin/Lys-C (V5071, Promega) in 50μM of ammonium bicarbonate. Samples were incubated at 37°C overnight. The following day, 0.5μL of 1M Tryspin/Lys-C was added and samples were incubated at 37°C for 1hr. For a final wash of digested peptides, 500μL of acetonitrile was added to each sample followed by incubation at room temperature for 10min and placement on a magnetic rack for washing in 200μL of acetonitrile. Peptides were eluted in 20μL 2% acetonitrile 0.2% formic acid for 10min at room temperature and dried using vacuum centrifugation (Vacufuge Plus, Eppendorf), then rehydrated prior to mass spectrometry in 0.1% formic acid and quantified using the micro-BCA assay (23235, Pierce). Samples were brought to 50ng/μL for all subsequent mass spectrometry analysis.

For human meningioma and meningeal samples, approximately 200ng per sample was analyzed on a TimsTOF Pro2 (Brucker) interfaced with an Easy-nLC 1200 HPLC (Thermo Scientific). Peptides were separated using a 15cm column (PepSep, 150μm ID, 1.5μm beads). HPLC buffer A contained 0.1% formic acid, and buffer B was comprised of 80% acetonitrile in 0.1% formic acid. The following gradient was used for peptide elution at a flow rate of 600nL/min: 3–27% B over 28min, a ramp to 40% B from 28–33min, and a ramp from 40–90% B from 33–34min, after which the system was held at 90% buffer B for 6min until the end of the acquisition (40min total length). For dog meningioma and meningeal samples, approximately 200ng per sample was analyzed on a TimsTOF Pro2 (Bruker) interfaced with an VanquishNeo HPLC (Thermo Scientific). Peptides were again separated using a 15cm column (PepSep, 150μm ID, 1.5μm beads), and separated in the same HPLC buffers A and B. The following gradient was used for peptide elution at a flow rate of 600 nL/min: 3–33% B over 33min, and a ramp to 95% B from 33–34min, after which the system was held at 90% buffer B for 6min until the end of the acquisition (40min total length). Data for both human and dog samples were acquired in DIA-PASEF mode using 24 windows of variable width ranging from 302.8–1199.7m/z and spanning an ion mobility range between 0.75 and 1.36Vs/cm2.

For human meningioma samples, a randomly selected cohort (n=53) was also analyzed in DDA-PASEF mode to generate an experiment-specific spectral library. The DDA-PASEF data was searched against the canonical and isoform human protein sequences from Uniprot (accessed 15 November 2023) using the default settings in Spectronaut Pulsar. For dog meningioma and meningeal samples, the gas-phase fractionation (7 fractions) was used to build a spectral library in Spectronaut Pulsar searching against the canonical sequences of the Canis lupus familiaris (Dog) proteome from Uniprot (proteome ID UP000002254, accessed 6 September 2024). In both cases, Pulsar search parameters included tryptic cleavage specificity, with 2 missed cleavages, fixed modification of carbamidomethylation on cysteine residues, variable modification of methionine oxidation and protein n-terminal acetylation, and a minimum peptide length of 7. The resulting spectral library peptide identifications were filtered to 1% FDR at the peptide and protein level. All DIA-PASEF samples were searched against the species appropriate spectral library using Spectronaut (version 18.5.231110.55695 for human, version 19.2.240905.62635 for dog) and filtered to a 1% FDR at both the peptide and protein level, with no data imputation.

### Mass spectrometry-based proteomic data filtration

Proteins with <2 unique peptides identified across 606 meningioma and 6 meningeal samples (TotalGroupMeasurement£2) were removed, resulting in 6,472 proteins for analysis. In individual samples for which only 1 unique peptide was identified for a particular protein (numFeature=1, TotalGroupMeasurement≥2), peptides were removed if that sample’s protein intensity value fell outside the minimum and maximum intensity range of the data set consisting of samples with ≥2 unique peptides for the same protein. Subsequently, proteins were filtered based on a minimum expression threshold across all samples, where proteins which were identified in <25% of samples were excluded, resulting in a total of 5,788 proteins. Finally, meningioma samples expressing <50% of the total number of identified proteins were removed (n=3, 0.5%), resulting in a dataset of 603 meningioma and 6 meningeal samples for analysis.

Batch effects across the seven 96-well plates that were used for proteomic analysis of meningioma samples were identified through uniform manifold approximation and projection (UMAP) and principal component analysis (PCA) visualizations. The ComBat function from the SVA package in R was used to correct for batch effects, and the quantitative matrix was adjusted for plate effects as specified in the metadata. After correcting for batch effects, ‘NA’ intensity values for proteins (indicating proteins that were not identified in a particular sample) were replaced with 0s, and the entire dataset was shifted by the absolute value of the most negative protein intensity to ensure all values were non-negative.

The same quality control and filtration pipeline was used for mass spectrometry-based proteomic analysis of data from dog meningioma (n=79) and meningeal (n=15) samples, resulting in a total of 6,697 proteins across all samples for analysis.

### Mass spectrometry-based proteomic clustering analysis

Clustering analyses were performed on both log transformed and non-log transformed data. UMAP and PCA were used to visualize potential associations between proteomic data and DNA methylation group^30,37,38^, DNA methylation subgroup^36,45^, integrated grade^43^, gene expression risk score^40,41,138,139^, World Health Organization (WHO) grade^49^, sex, DNA methylation family^31^, and integrated score^44^. K-means clustering was used to visualize k=2 through k=6 hypothetical meningioma proteomic clusters, and consensus clustering was performed using the ConsensusClusterPlus package^140^. The elbow method and silhouette analysis using the fviz_nbclust function from the factoextra package were used in an attempt to identify the optimal number of proteomic clusters.

The top 2000 proteins with the most variable intensities across all meningioma samples were selected for heatmap visualization, which was generated using the ComplexHeatmap package^141^ with both hierarchical and k-means clustering. To assess the robustness and consistency of clustering results, the same approaches were used on different preprocessed data and data subsets including non-log data, batch-corrected data using the limma package^142^, and on complete cases including only proteins with detectable intensities across all meningioma samples, without a clear or consistent number of hypothetical meningioma proteomic clusters using any approach.

### Mass spectrometry-based proteomic differential expression analysis

The edgeR package^143–145^ and a matrix that incorporated batch information were used for mass spectrometry-based proteomic differential expression analysis, which was performed with the lmFit and eBayes functions in the limma package^142^. Contrasts were defined for comparisons, such as patient sex, meningioma versus meningeal samples, and meningioma WHO grades or DNA methylation groups. Results were extracted using the topTable function, and significant results were filtered based on a p-value threshold of 0.05. Volcano plots were generated to visualize differential expression across groups using the EnhancedVolcano package.

### Protein-RNA expression correlation analysis

Filtered, batch-corrected, log_2_ transformed proteomic data were used for protein-RNA correlation analyses, which were performed on 446 meningioma samples with available proteomic and transcriptomic data from RNA sequencing^30,36^. The ggplot2, reshape2, and dplyr packages were used to merge and visualize protein and gene expression data^146–148^. R^2^, Pearson, and Spearman correlation coefficients were calculated for each protein-RNA pair using the stats and ggplot2 packages and the cor and cor.test functions.

### Ontology analysis

Protein and gene lists were analyzed for Gene Ontology (GO) Biological Processes (BP) enrichment using the org.Hs.eg.db and clusterProfiler packages in R^149^. The enrichGO function from the clusterProfiler package was used for the enrichment analysis, with parameters set to adjust p-values using the Benjamini-Hochberg (BH) method and cutoff values of 0.05 for both p- and q-values. Enriched GO terms were visualized using the ggplot2 and enrichplot packages.

### Cell culture

HEK293T cells (CRL-3216, ATCC), IOMM-Lee meningioma cells^150^ (CRL-3370, ATCC), CH157-MN meningioma cells^151^, BenMen meningioma cells^152^, and LN18 glioblastoma cells (NCB2108FY0116, Creative Biolabs) were cultured in DMEM (11960069, Life Technologies) supplemented with 10% FBS, 1x GlutaMAX (35050–061, Thermo Scientific). ID1654^87^, M10G^47^, and M13C^47^ patient-derived meningioma cells were cultured in 50% DMEM F12 (11320033, Life Technologies) and 50% Neurobasal Media (21103049, Life Technologies) supplemented with 5% FBS, 1x GlutaMAX, N2 Neuroplex supplement (400163, Gemini), B-27 supplement without vitamin A (12587010, Gibco), EGF 20ng/ml (236EG200, R&D Systems), and FGF basic/FGF2 20ng/ml (PRD23350, R&D Systems). T47D breast cancer cells were cultured in RPMI (11875093, Thermo Scientific) supplemented with 10% FBS and human recombinant insulin 0.01mg/mL (12585014, Thermo Scientific). MCF7 breast cancer cells were cultured in EMEM (M4655, Sigma) supplemented with 10% FBS, human recombinant insulin 0.01mg/mL (12585014, Thermo Scientific), and 1% non-essential amino acids (11140050, Thermo Scientific). Cell lines were confirmed mycoplasma-free at regular intervals during experimentation and were validated using SSTR profiling and DNA methylation profiling.

CH157-MN^dCas9-KRAB^, IOMM-Lee^dCas9-Zim3^, BenMen^dCas9-Zim3^, and M10G^dCas9-Zim3^ meningioma cells stably expressing BFP+ CRISPR interference (CRISPRi) machinery were generated by viral transduction. Lentiviral particles were produced from HEK293T cells transfected with psPAX2 (12260, Addgene) and pMD2.G (12259, Addgene) plasmids, and either pMH0001 (UCOE-SFFV-dCas9-BFP-KRAB) or pJB108 (dCas9-Zim3-KRAB-BFP) using TransIT-Lenti Transfection reagent (6605, Mirus) and polybrene 8μg/mL (TR-1003-G, EMD Millipore). Transduced cells were isolated through double selection of the top 10% of BFP+ cells using fluorescence activated cell sorting. CRISPRi activity was validated using competitive growth assays to measure depletion of cells expressing RFP+ sgRNAs targeting common essential genes versus non-targeted control sgRNAs (sgNTC). sgRNA protospacer sequences suppressing *PGRMC1* (5’-GCACTCGCTCGCTCAGAGGG-3’)*, PGRMC2* (5’-GCTGGGGGCCTACCGGCTGT-3’), or *PGR* (5’-GTCCCTCCTCCTGGAGACGG-3’) were ligated into the pCRISPRia-v2 vector83 (848320, Addgene), and lentivirus was generated as described above. Meningioma cells stably expressing CRISPRi machinery and sgRNAs were selected to purity using puromycin for 3 days. Gene suppression was confirmed using QPCR and immunoblots as described below.

To generate meningioma cell lines stably expressing progesterone receptor (PR), Src-binding mutant PR (P422A, P423A, and P426A), PGRMC1, or PGRMC1^D120G^, pLVX-Puro plasmids were generated containing each gene of interest. Lentivirus was produced as described above, and meningioma cells were transduced with lentiviral particles for each expression construct and selected to purity using puromycin for 3 days. To generate meningioma cell lines stably expressing 3x-FLAG tagged FXR1 or RBM39, pLVX-Puro plasmids were generated as described for N-terminally tagged FXR1 and C-terminally tagged RBM39, which was selected for least predicted interference with each protein’s functional domains. Cells were transduced and selected as described above, and gene expression was confirmed using QPCR and immunoblots as described below.

To suppress *FXR1* or *RBM39* in meningioma cells, ON-Targetplus siRNA (Dharmacon) containing a mixture of 4 siRNAs per target versus non-targeted control siRNAs were delivered using Dharmafect 4 Transfection Reagent at the maximum concentration recommended by the manufacturer, which was empirically determined to achieve the highest knockdown efficiency in meningioma cells compared against Dharmafect 1, 2, or 3 Transfection Reagent. Gene suppression was confirmed using QPCR as described below.

Given the impact of sex-specific serum hormones on meningioma cell proliferation (Extended Data Fig. 10a), cells were transitioned to media containing charcoal-stripped FBS for at least 1 day prior to all genetic or pharmacologic experiments. Progesterone (P4, 8783, Sigma-Aldrich) was solubilized to 10mM in ethanol, and cells were treated with progesterone 1μM as previously reported for activation of PGRMC1 signaling in cell culture^153^, or control media containing an equal volume of ethanol in charcoal stripped media. Medroxyprogesterone acetate (MPA, 6013, Sigma-Aldrich) was solubilized to 100mM in acetone, diluted to 10mM in ethanol, and cells were treated with MPA 1μM (which achieved the greatest empiric meningioma cell proliferation and *PGR* expression in dose-titration experiments) or control media containing an equal volume of 9:1 ethanol:acetone in charcoal stripped media. Cyproterone acetate (CPA, 16622, Cayman) was solubilized in DMSO to 10mM, and cells were treated with CPA 1μM (an equivalent dose to progesterone treatment, as CPA and progesterone have similar binding affinities for PR^154^) or control media containing an equal volume of DMSO in charcoal stripped media. AG205 (1487, Sigma-Aldrich) was solubilized to 5mM in DMSO, and cells were treated with AG205 10μM (which achieved the greatest empiric inhibition of *PGR* expression in surviving meningioma cells in dose-titration experiments) or control media containing an equal volume of DMSO in charcoal stripped media. Indisulam (13650, Medchem Express) was solubilized to 10mM in DMSO, and cells were treated with indisulam 20μM (which achieved the greatest reduction in RBM39 protein expression while maintaining meningioma cell viability >90% over 2 days) or control media containing an equal volume of DMSO in charcoal stripped media. Testosterone (T1500, Sigma-Aldrich) was solubilized to 10mM in ethanol, and cells were treated with testosterone 10μM (which achieved the greatest empiric inhibition of meningioma cell proliferation in response to MPA) or control media containing an equal volume of ethanol in charcoal stripped media.

### CRISPRi screening

A custom CRISPRi screening library for RNA binding/splicing/modifying factors was generated using proteomic data from human meningiomas (n=603). All proteins within RNA biology-related ontology terms from the proteomic atlas (Fig. 1d, g, Extended Data Fig. 1j) were queried in the published literature, and candidates were included in the sgRNA library only if RNA splicing/binding/modifying function was described in at least 2 publications. Common essential ribosome components were excluded. This approach yielded a list of 447 RNA processing genes. 29 meningioma-specific tumor suppressor genes or oncogenes, including steroid hormone receptors and epigenetic regulators, from the published literature and 30 common essentials were included as positive controls. In Supplementary Table 7, the “Transcript” column identifies the transcription start site targeted by each sgRNA and “Selection Rank” column rates the confidence of each sgRNA as predicted by the CRISPRi-v2.1 algorithm, with 1 being the highest confidence sgRNA for each gene. Five to ten sgRNAs per gene and 250 sgNTCs were selected from the hCRISPRi v2.1 library^155^ and cloned into pLG01-mCherry. Pooled lentivirus was produced by transfection of HEK293T cells with the sgRNA library as described above, and M10G^dCas9-Zim3^ meningioma cells were transduced to an MOI of 0.26 and >1000x coverage was maintained throughout the screen. Three replicates were performed for each arm of the screen, cells were selected for sgRNA expression using puromycin for 2 days, and time=0 (T0) cells were collected and frozen 5 days after sgRNA transduction. The remaining cells were divided and cultured as described above in MPA 1μM or vehicle control. Cells from MPA and control arms were collected after 10 passages and genomic DNA was isolated from MPA, control, and T0 replicates (n=9) using the NucleoSpin Blood XL Kit (740,950.50, Machery-Nagel). sgRNA sequencing libraries were prepared using NEBNext Ultra II Q5 PCR MasterMix (M0544L, New England Biolabs) and sequenced on an Illumina NextSeq-500 with 1.5B read coverage.

Raw FASTQ files were processed using the model-based analysis of genome-wide CRISPR/Cas9 knockout (MAGeCK) algorithm^156^. sgRNA sequences were aligned to the reference library using the MAGeCK count function, and library normalization was performed using the median normalization method, with sgNTCs specified as controls. Differential sgRNA abundance was assessed using the MAGeCK test module, and gene-level enrichment and depletion scores were calculated using the MAGeCK robust rank aggregation (RRA) algorithm to provide beta values representing log-fold change measurements compared with baseline non-targeting controls.

### RNA processing risk score development and validation

Regression analysis to identify protein predictors of overall survival (OS) from meningioma was performed using human meningioma samples with available proteomic mass spectrometry and clinical follow-up data (n=601). Proteomic mass spectrometry expression of RNA processing proteins in a randomly selected discovery cohort (n=425, 70.7%) were used as inputs for a LASSO regularized Cox regression model in the *glmnet* package in R that was trained using 10-fold cross-validation and the c-index of OS as the target endpoint to develop a risk prediction score that was comprised of 26 proteins. The resulting RNA processing risk score was linearly rescaled from 0 to 1 and tested for prediction of OS and progression free survival in a randomly selected independent validation cohort that was not used for RNA processing risk score development or training (n=176, 29.3%). A nested rank statistic was used for delineation of risk groups (low, intermediate, and high) with cutoffs specific for OS (0.34 to separate low- and intermediate-risk, 0.60 to separate intermediate- and high-risk) that were used for Kaplan-Meier visualization.

### Cross-linking immunoprecipitation (CLIP) sequencing and analysis

Biological replicates (n=2 per condition) consisting of four 15-cm plates at 90% confluence of CH157-MN meningioma cells stably expressing FXR1–3x-FLAG or 3x-FLAG-RBM39 were treated with MPA, progesterone, or vehicle control as described above for 4hrs. Cells were washed with PBS and crosslinked with 400 mJ/cm^2^ 254nm UV, as previously described^157^. Crosslinked cells were lysed on ice in low salt buffer (1x PBS, 0.1% SDS, 0.5% sodium deoxycholate, 0.5% IGEPAL CA-630) supplemented with Ribolock RNase Inhibitor (EO0381, Thermo Scientific) and 1x Protease Inhibitor (78425, Thermo Scientific). Lysate was treated with RQ1 DNase (M6101, Promega) at 37°C for 5min, and to fragment RNA, lysates were treated with RNase A (EN0531, Thermo Scientific) and RNase I (AM2294, Invitrogen) under high (RNase A 1:3,000 + RNase I 1:100) and low (RNase A 1:15,000 + RNase I 1:500) digestion conditions at 37°C for 5min. High- and low-digested lysates were combined and clarified by centrifugation at 20,000rcf at 4°C for 20min. Clarified lysate was transferred to anti-Flag M2 beads (M8823, Sigma), and rotated end-over-end at 4°C for 3hrs. Beads were washed twice with low salt buffer, high salt buffer (5x PBS, 0.1% SDS, 0.5% sodium deoxycholate, 0.5% IGEPAL CA-630), and PNK buffer (50mM Tris pH 7.4, 10mM MgCl_2_, 0.5% IGEPAL CA-630).

RNA 3′ ends were dephosphorylated on anti-Flag M2 beads using T4 polynucleotide kinase (M0201L, New England Biolabs) in 1x PNK buffer at 37°C for 20min with orbital shaking. Beads were washed and RNA was subjected to limited poly(A) tailing using yeast poly(A) polymerase (RNT-006-L, Jena Bioscience) with ATP at 22°C for 5min. RNA was azido labeled using 3′-azido-3′-deoxyuridine-5′-triphosphate (NU-992, Jena Bioscience) at at 37°C for 20 min. Following washing, poly(A) and azido-modified RNA was conjugated to IRDye 800CW DBCO (LI-COR Biosciences) using copper-free click chemistry at 22°C for 30min with protection from light. RNA-protein complexes were eluted in 1x NuPAGE LDS sample buffer (NP0007, Invitrogen) supplemented with 50mM DTT and heated at 75°C for 10 min.

Eluted RNA-protein complexes were resolved on 4–12% Bis-Tris NuPAGE gels (NP0322BOX, Invitrogen) in MOPS running buffer (NP0001, Invitrogen) at 180V at 4°C for 60–90min. IR-labeled protein standards (928–60000, LI-COR) were included, and RNA-protein complexes were transferred to BA-85 Protran nitrocellulose membranes (10402525, Cytiva) using 1x NuPAGE transfer buffer (NP00061, Invitrogen) containing 10% ethanol at 30V for 75min. Membranes were briefly rinsed in PBS and imaged on a LI-COR Odyssey infrared imaging system, and the relevant regions for FXR1 and RBM39 (60–75kDa) were cut from membranes. Excised nitrocellulose fragments were incubated in Proteinase K digestion buffer (AM2548, Invitrogen) at 55°C for 45min with shaking, and RNA in supernatant was collected and adjusted to 500mM NaCl to promote hybridization to oligo(dT)25 magnetic beads (61002, Invitrogen and S1419S, New England Biolabs). RNA was captured by incubation at 25°C for 20min, washed with high-salt buffer and PBS, and eluted in TE buffer at 50°C for 5min.

cDNA libraries were generated from captured RNAs using the SMARTer smRNA-seq Kit for Illumina (635031, Takara Bio). Eluted RNA was annealed to a UMI-containing oligo(dT) RT primer (custom oAN491, IDT), followed by reverse transcription using PrimeScript RT. PCR amplification was performed using SeqAmp DNA Polymerase (638509, Takara Bio) and SeqAmp CB PCR Buffer (638526, Takara Bio) with indexed forward primers and a universal reverse primer (oAN426). Cycling conditions were 98°C for 1min, followed by 25 cycles of 98°C for 10sec, 60°C for 5sec, and 68°C for 10sec.

PCR products were purified using magnetic SPRI beads (D4084–50, Zymo Research) at a 1.1x ratio and eluted in nuclease-free water. Library size distribution and concentration were assessed using High Sensitivity DNA TapeStation (Agilent), and gel purification was performed using 8% TBE NuPAGE gels (EC62152BOX, Invitrogen) followed by diffusion-based elution and ethanol precipitation. Libraries were sequenced as single-end 100bp reads on an Illumina NovaSeqX with 10B read coverage and demultiplexing at i5.

Raw sequencing data from FXR1 and RBM39 CLIP sequencing experiments were processed by appending unique molecular identifiers (UMIs) to reads using UMI-tools^158^. Processed reads were analyzed using the CLIP Tool Kit (CTK) pipeline^159^, which includes adapter trimming with cutadapt^160^, alignment to the human reference genome (hg38) using bwa^161^, PCR deduplication with UMICollapse^162^, and peak calling. Biological replicates were pooled prior to crosslinking-induced mutation sites (CIMS) analysis to identify nucleotide deletions indicative of direct protein-RNA crosslinking. High-confidence RNA binding protein sites (FDR<10%) were stored in browser extensible data (BED) format and annotated to genomic features using bedtools intersect^163^ and the Gencode v47 reference genome.

### Long-read RNA sequencing and analysis

RNA was extracted from frozen meningioma samples (n=93) using the RNeasy Mini Kit (74106, QIAGEN) and those with available short-read RNA sequencing data^30,36^ were used for analysis (n=84). Iso-Seq libraries were constructed using the Iso-Seq Express 2.0 Kit (103-071-500, Pac Bio) and SMRTbell Prep Kit 3.0 (103-381-200, Pac Bio) and sequenced on a Revio using a total of 12 SMRT Cells. Movies were acquired for 30hrs with base kinetics included and consensus mode set to molecule. Short- and long-read RNA sequencing data were processed using the multi-platform aggregation and quantification of transcripts (MPAQT) pipeline, which integrates complementary sequencing modalities to achieve accurate transcript isoform-level quantification^164^. In brief, the MPAQT pipeline involves reference index generation, sequencing modality-specific preprocessing, and joint transcript quantification based on both short-read and long-read data. All analyses were performed using the human reference transcriptome and genome annotation in Gencode v47^165^. Previously published meningioma short-read RNA sequencing data were preprocessed uniformly using adapter trimming and quality filtration in cutadapt^160^ to remove TruSeq adapters and low-quality bases. Filtered reads were pseudoaligned to the transcriptome using kallisto^166^ to generate pseudoalignment and equivalence class (EC) matrices for downstream integration. Meningioma long-read RNA sequencing data were preprocessed using the PacBio Iso-Seq pipeline. Raw BAM files were demultiplexed by PCR and SMRTbell barcodes using lima, and full-length non-chimeric (FLNC) reads were generated using the isoseq refine function, which performs poly(A) tail trimming and concatemer removal, ensuring capture of complete transcript structures. FLNC reads were clustered using the isoseq cluster2 function to collapse reads representing the same transcript. Clustered reads were converted from BAM to FASTQ format using samtools^167^ and aligned to the human reference genome (hg38) with minimap2^168^. Isoform quantification was generated using bambu^169^ in quantification mode to obtain transcript-level abundance estimates. Short-read kallisto BUS files and EC matrices and long-read bambu transcript quantifications were integrated using MPAQT to generate accurate isoform-level transcript expression quantifications across all samples. Transcript abundances (TPM) from the MPAQT output were analyzed using SUPPA2^170^ to identify and quantify local alternative splicing (AS) events. Events were generated from the Gencode v47 annotation using the generateEvents function, which captures 7 AS event types: skipping exon (SE), alternative 5’ and 3’ splice sites (A5/A3), mutually exclusive exons (MXE), retained intron (RI), and alternative first and last exons (AF/AL). Percent spliced-in (PSI) values were calculated for each event across all samples using the psiPerEvent function, and differential AS analysis was performed using the diffSplice command to identify differential AS events between clinical subgroups. Significant events were defined as |ΔPSI|>0.1 and p-value<0.05. The 2,000 most variable AS events (ranked by z-score variance across samples) were displayed using the ComplexHeatmap and circlize packages in R^141^. Ontology analysis was performed for the top 2,000 most variable AS events.

### Actinomycin D assays

For RNA stability experiments, M13C meningioma cells and T74D and MCF7 breast cancer cells were grown in MPA as described above and transfected with siRNAs targeting *FXR1* or non-targeted control siRNAs as described above 2 days prior to treatment with actinomycin D 5μM (A1410, Siga-Aldrich), as previously described^171^, and 1 day prior to treatment with indisulam 20μM. After 0, 4, 8, 12, and 16hrs of combination treatment with MPA and actinomycin D ± indisulam, cells from each timepoint were harvested simultaneously and RNA was extracted for QPCR as described below.

### Quantitative reverse-transcriptase polymerase chain reaction (QPCR)

RNA was extracted from cultured meningioma cells or meningioma xenografts using the RNeasy Mini Kit (74106, QIAGEN). cDNA was synthesized using the iScript cDNA Synthesis Kit (1708891, Bio-Rad). QPCR was performed using PowerUp SYBR Green Master Mix (A25918, Thermo Scientific) on a QuantStudio 6 Flex Real-Time PCR system (Life Technologies) using the following forward and reverse primers: *RBM39*-F CAATGCTTGAGGCTCCTTACA, *RMB39*-R TCCGTTCCTTACTTTTGCTTCTC, *FXR1*-F GAGAGAAGATTTAATGGGCCTGG, *FXR1*-R GCTCAATGGCGGTAACTCCA, *PGR*-F GCACGAGTTTGATGCCAGAGA, *PGR*-R CTGCGACGGCAATTTACTGA, *PGRMC1*-F GGGCTGCTGCATGAGATTTTC, *PGRMC1*-R CCGCGCACGATCTTGTAGA, *PGRMC2*-F ATTTGCTGGTAGGGATGCCTC, *PGRMC2*-R TCGAACACTCTCCATTTGTACTG, *GAPDH*-F GTCTCCTCTGACTTCAACAGCG, *GAPDH*-R ACCACCCTGTTGCTGTAGCCAA, 5S-F GCCATACCACCCTGAACG, and 5S-R GGTATTCCCAGGCGGTCT). QPCR data were analyzed using the ΔΔCt method relative to *GAPDH* expression in each sample.

### Immunoblotting

For immunoblotting from cultured cells, samples were lysed in ice-cold RIPA buffer (156034, Abcam) containing protease and phosphatase inhibitors (A32961, Thermo Scientific). Samples were vortexed at maximum speed for 15sec, rotated at 4°C for 15min, vortexed again at maximum speed for 15sec, and centrifuged at 15,000rcf at 4°C for 15min. Protein concentration was quantified using the BCA assay (23225, Thermo Scientific).

For immunoblotting from human tumor samples or xenografts, frozen tissue was lysed in 2% SDS, 100mM pH6.8 Tris-HCL containing protease and phosphatase inhibitors (A32961, Thermo Scientific) at 4°C. Samples were dissociated using a TissueLyserII (QIAGEN) at 30Hz for 3min. Samples were subsequently sonicated at 40% Ampl on ice for 15sec (QSonica Q700), rotated at 4°C for 15min, and centrifuged at 15,000rcf at 4°C for 15min. Protein concentration was quantified using the BCA assay (23225, Thermo Scientific).

For subcellular fractionation and immunoblotting, cell cultures were fractionated using the Subcellular Protein Fractionation Kit (78840, Thermo Scientific). Protein concentration was quantified using the BCA assay (23225, Thermo Scientific) and the Bradford assay (23200, Thermo Scientific).

To prepare samples for immunoblotting, 20μg of protein lysate from cultured cells or tumor samples, or 5μg of protein lysate from subcellular fractionation experiments, were boiled for 15min in Laemmli reducing buffer (J61337-AD, Thermo Scientific). Proteins were separated on 4–15% TGX precast gels (5671084, Bio-Rad), and transferred onto ImmunBlot PVDF membrane (1620177, Bio-Rad). Membranes were blocked in 5% BSA in TBST (tris-buffered saline, 0.1% Tween-20), incubated in primary antibodies, washed, and incubated in secondary antibodies. Membranes were subjected to immunoblot analysis using Pierce ECL substrate (32209, Thermo Fischer Scientific). Primary antibodies recognizing PR (3172, Cell Signaling, 1:500 or 2765, Abcam, 1:500), PGRMC1 (194879, Abcam, 1:1000), RBM39 (21339, Proteintech, 1:1000), FXR1 (245624, Abcam, 1:1000), GAPDH (8245, Abcam, 1:2000), tubulin (6046, Abcam, 1:2000), calnexin (2679, Cell Signaling, 1:2000), RB (9309, Cell Signaling, 1:1000), HH3 (22481, Abcam, 1:5000), pPR (Ser345) (12783, Cell Signaling, 1:1000), MEK (9122, Cell Signaling, 1:1000), pMEK (9154, Cell Signaling, 1:1000), ERK1/2 (4695, Cell Signaling, 1:1000), pERK1/2 (9101, Cell Signaling, 1:1000), cyclin D1 (8396, Santa Cruz, 1:1000), pPAX (69363, Cell Signaling, 1:1000), pEGFR (11862, Cell Signaling, 1:1000), pAKT (S473) (9271, Cell Signaling, 1:1000), and secondary antibodies recognizing mouse (7076, Cell Signaling, 1:2,000) or rabbit (7074, Cell Signaling, 1:2,000) epitopes, were used. Secondary antibodies used for immunoblotting after immunoprecipitation, described below, were VeriBlot (131366, Abcam, 1:1000) for PGRMC1 and anti-light chain specific anti-rabbit IgG (99697, Abcam, 1:1000) for FXR1 and RBM39.

### Immunoprecipitation and immunoprecipitation mass spectrometry (IP-MS)

Meningioma cell lines were grown to 90% confluence in 10cm plates and treated as described above. Cells were lysed in 800μL buffer containing 50mM HEPES pH7.5 (7365-45-9, Thermo Scientific), 150mM NaCl (AM9760G, Invitrogen), 1% NP-40 (85124, Thermo Scientific), 1mM EDTA (AM9260G, Invitrogen), 5% glycerol (A16205.0F, Thermo Scientific), and protease and phosphatase inhibitors (A32961, Thermo Scientific) at 4°C. Samples were vortexed at maximum speed for 15sec, rotated at 4°C for 15min, vortexed again at maximum speed for 15sec, and centrifuged at 15,000rcf at 4°C for 15min. 10ug of antibodies recognizing FXR1 (245624, Abcam), RBM39 (21339, Proteintech), PGRMC1 (194879, Abcam), or control IgG (2027, Santa Cruz) were added to the supernatant of samples. Samples were rotated overnight at 4°C followed by addition of 10μL of PBS-washed Protein A/G magnetic beads (88802, Thermo Scientific) and rotation at 4°C for 1hr. Beads were isolated on a magnetic rack and washed 3 times for 5min each in lysis buffer, followed by one 5min wash in PBS. For IP-MS, samples were eluted in 100μL of 2% SDS 100mM pH6.8 Tris-HCL containing 10mM DTT at 55°C for 15min, then processed for mass spectrometry. For immunoblotting, samples were eluted in 25μL of 1x Laemmli-SDS sample buffer (111NR, Boston BioProducts) containing 100mM DTT at 65°C for 15min.

For IP-MS data acquisition and analysis, samples were analyzed on a Bruker timsTOF Pro 2 mass spectrometer coupled to a Vanquish Neo UHPLC system (Thermo Fisher Scientific). Peptides were separated on a 15cm analytical PepSep column (150μm inner diameter, 1.5μm particle size). Mobile phase A consisted of 0.1% formic acid in water, and mobile phase B consisted of 80% acetonitrile with 0.1% formic acid. Peptides were eluted at a flow rate of 600nL/min using the following gradient: 3–33% B over 33min, increased to 95% B from 33min to 34min, followed by a hold at 90% B for 6min, for a total run time of 40min. Data were acquired in data-dependent acquisition parallel accumulation-serial fragmentation (DDA-PASEF) mode using Bruker default settings. Mass spectrometry acquisition ranged from m/z 100 to 1700. The ion mobility range was set from 1/K_0_ = 1.3 to 0.85 V·s/cm^2^. Ion accumulation and ramp times in the TIMS analyzer were both set to 100msec, resulting in a total cycle time of approximately 0.53sec. Collision energy was applied as a function of ion mobility, with 59eV at 1/K_0_ = 1.6 V·s/cm^2^ and 20eV at 1/K_0_ = 0.6 V·s/cm^2^. TIMS calibration was performed using Agilent ESI LC/MS tuning mix ions with reference values (m/z, 1/K_0_): 622.0289 (0.9848 V·s/cm^2^), 922.0097 (1.1895 V·s/cm^2^), and 1221.9906 (1.3820 V·s/cm^2^). Raw data were processed using FragPipe^172^. Spectra were searched against the UniProt human canonical protein database (20,416 entries, downloaded February 23, 2026). Search parameters included trypsin and Lys-C specificity with up to two missed cleavages. Carbamidomethylation of cysteine was set as a fixed modification, while methionine oxidation and protein N-terminal acetylation were set as variable modifications. A minimum peptide length of 7 amino acids was required. Peptide and protein identifications were filtered to a 1% false discovery rate. Protein-protein interaction scoring was performed using SAINTexpress^173^ within FragPipe, with IgG samples used as controls. Data visualization and statistical analyses were carried out in R using the artMS and MSstats packages^174^.

For analysis of FXR1 and RBM39 IP-MS data (Supplementary Table 14), proteins with >2 peptides, >2 spectral count, and >40,000 total average intensity for either vehicle control or MPA conditions were retained for analysis. Proteins with enrichment in average intensity over IgG control of greater than 0.15 in any samples, or with total intensity in IgG samples over 200,000, were excluded from analysis. Proteins that did not appear in all 3 replicates for at least one treatment condition were excluded from analysis.

For analysis of PGRMC1 IP-MS data, proteins with >2 peptides and >2 spectral count were retained for analysis. Proteins that appeared in IgG control samples were excluded from analysis. For analysis of IP-MS data from CH157-MN meningioma cells (Supplementary Table 15), proteins that did not appear in 2 replicates for at least 1 treatment condition were excluded from analysis. For analysis of PGRMC1 IP-MS data across meningioma cell lines (Extended Data Fig. 8a), proteins that appeared in at least 2 of 3 cell lines were retained for analysis. To determine relative intensity of proteins across meningioma cell lines (CH157-MN, IOMM-Lee, ID1654) with versus without MPA treatment, the total intensity of each protein was divided by the total intensity of PGRMC1 in each sample. The higher intensity condition between treatments was normalized to 1 for each protein, and the lower intensity condition between treatments was represented as a fraction of that intensity.

### Structural modeling

AlphaFold3 (v3.0.1) and its reference databases were installed and run locally, and interactions were modeled for PGRMC1 (O00264–1), FXR1 (P51114–1), and/or RBM39 (Q14498–1) with or without progesterone, MPA, CPA, nomegestrol acetate (NOMAC), chlormadinone acetate (CMA), AG205, or testosterone. AlphaFold3 was run with seeds = [1,2,3,4,5] and 5 models output per seed. Where applicable, PGRMC1 was trimmed to residues 60–195 to remove the partially disordered and flexible transmembrane domain to prevent confounding effects on affinity scoring, and FXR1 was trimmed to residues 1–385 and RBM39 was trimmed to residues 126–508 to remove disordered domains. Boltz and Chai-1 were also installed and run locally to corroborate AlphaFold3 modeling results for the aforementioned proteins and small molecules. Boltz2 was run with default settings except for -- use_msa_server, --recycling_steps=10, --diffusion_samples=25, and --diffusion_samples_affinity=25. Runs were performed with and without physical potentials (--use_potentials). Chai-1 was run with default settings except for the following: --num-trunk-samples 5, --num-trunk-recycles 4, --seed 1, --num-diffn-samples 5. Minimum backbone distances between the cytochrome *b*_*5*_-like domain of PGRMC1 (residues 101–165) and the FXR1 KH2 domain (residues 282–340) and Tud1 and Tud2 domains (residues 1–127) was determined from each algorithm’s output structures.

### Immunofluorescence microscopy and analysis

Meningioma cell cultures for immunofluorescence staining were grown on glass cover slips and fixed in 4% PFA in PBS for 8min, washed in PBS, and blocked for 1hr in 5% donkey serum and 0.1% Triton-X100 in PBS. Cells were stained with primary antibodies recognizing PGRMC1 (194879, Abcam, 1:1000), lamin A/C (238303, Abcam, 1:500), GORASP2 (CL488–66627, Proteintech, 1:250), calnexin (MA3–027, Invitrogen, 1:250), or pPAX (69363, Cell Signaling, 1:500) overnight at 4°C and labeled with rabbit Alexa Fluor secondary antibody (A21206, Thermo Scientific, 1:1,000) and Hoechst 33342 (H1399, Thermo Scientific, 1:2000) to mark DNA for 1hr at room temperature prior to mounting and imaging. For actin imaging, cells were stained with phalloidin conjugated to Alexa Fluor 647 (A22287, Thermo Scientific, 1:50) for 15min at room temperature. Samples were imaged on an LSM800 confocal laser scanning microscope with Airyscan (Zeiss) and images were analyzed using ImageJ.

### Clonogenic assays

For clonogenic assays, 2000 cells in 10cm plates or 500 cells in 6-well plates were seeded in triplicate for each experimental condition and grown for 10 days. For MPA, progesterone, AG205, and testosterone treatments, as well as vehicle controls, media was changed every 2 days. Cells were fixed in methanol for 30min, washed and stained in 0.01% crystal violet (C6158, Sigma-Aldrich) for 3hrs, washed three times in distilled water, and dried overnight.

### Mice

Mouse meningioma xenograft experiments were performed by implanting 3 million CH157-MN or IOMM-Lee cells into the flank of 4- to 6-week-old female or male *Nu/Nu* mice (Harlan Sprague Dawley). Of note, meningiomas are not protected by blood brain barrier^124^. For castration of male mice, 5 days prior to xenograft implantation mice were anesthetized and a small midline incision was made in the ventral scrotum to exteriorize the testes, which were ligated and removed before closing the incision with absorbable sutures. MPA was delivered to mice via intraperitoneal (IP) injection at a daily dose of 10mg/kg in sterile PBS. AG205 was solubilized to 50mM in DMSO, diluted to 2mg/mL in PBS, and delivered to mice via IP injection at a daily dose of 10mg/kg starting at the time of tumor cell implantation. Indisulam was solubilized to 25mg/mL in DMSO, diluted 5mg/mL in PBS, and delivered to mice via IP injection at a daily dose of 25mg/kg after the appearance of visible tumors. Estrogen was delivered to mice via IP injection at a daily dose of 1mg. Testosterone was delivered to female mice via implantation of time-release subcutaneous pellets (5mg/60days, SA-211, Innovative Research)^175^. For pellet implantation, mice were anesthetized and a small incision was made in the dorsal neck where the pellet was inserted before closing the incision with absorbable sutures. Tumor volume was measured using calipers (tumor volume = diameter 1 × (diameter 1)/2 × diameter 2, where diameter 1 < diameter 2). For Kaplan-Meier survival analyses, events were recorded when tumors reached protocol-defined endpoints of >2,000 mm^2^ or >50% ulceration by surface area, or when mice developed mobility or physiologic impairment from tumor burden or lost >15% of body weight due to tumor burden.

### Histology, immunohistochemistry, microscopy and analysis

Deparaffinization and rehydration of 5μm formalin-fixed paraffin-embedded (FFPE) tumor tissue sections and hematoxylin and eosin (H&E) staining were performed using standard clinically validated procedures. After fixation, tissue was processed, embedded in paraffin, sectioned (5μm), and stored at −20°C prior to use. Slides were stained with H&E or immunostained using a Discovery Ultra autostainer (Ventana Medical Systems).

For immunohistochemical (IHC) signal detection, the Multimer HRP kit followed by the DAB detection kit was used (Ventana Medical Systems), and appropriate positive and negative controls were included. Following antigen retrieval with CC1 (Ventana Medical Systems) for 32min, sections were stained with primary antibodies recognizing cleaved caspase-3 (CC3, 9661, Cell Signaling Technology, 1:100), IBA1 (019–19741, Fujifilm Wako Chemicals, 1:500) or PR (1E2, Ventana Roche CONFIRM, undiluted).

For immunofluorescence signal detection, following antigen retrieval with the EZPrep and CC1 kits for 40min (Ventana Medical Systems), the Multimer HRP kit (Roche Diagnostics) was used to stain for FITC (760–232), Rhodamine (760–233), and CY5 (760–238) labeling of PR (1E2, Ventana Roche CONFIRM, undiluted), PGRMC1 (194879, Abcam, 1:2500), and somatostatin receptor 2 (SSTR2, UMB1, Abcam, 1:100). Following staining, slides were counterstained with DAPI (Sigma Aldrich) at 5μg/ml in PBS for 15min, mounted with ProLong Gold Antifade Mountant (Invitrogen), and stored at 4°C until imaging. Reagents from Roche Diagnostics included Omap anti-Rabbit HRP (760–4311), and Discovery Umap anti-rabbit HRP (760–4315).

Quantitative analyses of H&E and IHC slides was performed by a neuropathologist using QuPath-0.5.1^176^. Mitotic figures were quantified by manual count across a total area of 1.6mm^2^ (10 high powered field equivalents) in regions of tumor with highest apparent mitotic activity not corresponding to an area of necrosis or frank inflammation in accordance with recent meningioma grading recommendations^50^. Quantification of cleaved caspase 3 positive cells was performed by manual count across a total area of 1mm^2^ in regions of tumor with highest apparent activity not corresponding to an area of necrosis. Quantification of the percent of IBA1 positive cells was performed by dividing a manual count of IBA1 positive cells by an automated count of total cells across a total area of 0.16mm^2^ (1 high powered field equivalent) in a region of tumor with highest apparent activity not corresponding to a region of necrosis. Automated cell counting was performed using the cell detection function in QuPath with default settings.

FFPE meningioma samples for spatial transcriptomic experiments were obtained from 6mm cores sectioned at 4μm thickness. H&E staining was performed using standard clinically validated procedures, and IHC for PR (RTU, Ventana Ultra, 1E2) was performed using a VENTANA Benchmark Ultra stainer (Roche Diagnostics). H&E and IHC sections were imaged using a Leica Aperio GT 450 microscope and a 40x objective. Images were analyzed using Aperio ImageScope software.

### Patient testosterone and meningioma volume analysis

The University of California San Francisco electronic medical record was queried to identify patients with meningioma diagnosis by ICD code and any history of serum testosterone laboratory testing. Patient records were individually reviewed, and patients with serum total testosterone laboratory test(s) within 12 months of brain imaging revealing meningioma diagnosis and before any treatment for meningioma were included. Patients were excluded if head imaging was not available for review. Patients were also excluded if the only testosterone laboratory test available occurred after meningioma diagnosis and after medical therapy that could influence testosterone level (*e.g*. androgen deprivation therapy, testosterone replacement therapy), such that baseline serum testosterone level at the time of or preceding meningioma diagnosis could not be determined. Total testosterone values in ng/dL were recorded along with other pertinent clinical information, such as patient age and sex, history of androgen deprivation therapy prior to meningioma diagnosis, indication for testosterone testing, meningioma location and WHO grade, and whether meningioma was diagnosed incidentally or in the setting of neurological symptoms. Meningiomas were delineated on head imaging for volumetric assessment by a radiation oncologist using MIM. Meningioma volume and serum testosterone levels were compared between groups defined by patient age, sex, and history of androgen deprivation therapy.

### H3K27Ac chromatin immunoprecipitation (ChIP) sequencing analysis, and PR ChIP sequencing and analysis

Previously published meningioma H3K27Ac ChIP sequencing data^32^ were re-analyzed, and ChIP for PR was performed using the SimpleChIP Plus Sonciation Kit (56383, Cell Signaling). CH157-MN meningioma cells were seeded at a density of 10 million cells per 15cm plate in triplicate and were treated with MPA 1μM or vehicle control for 4hrs. For this analysis, meningioma cells expressing only PR isoform B or both PR isoforms A and B were used to establish which downstream targets were bound by isoform B, and which are specific to isoform A. PR expression constructs were generated and expressed as described above. Twenty-four hours after seeding, the number of cells on one 15cm plate was counted and cell viability was confirmed as >90%. Cells were fixed with 1% methanol-free formaldehyde (PI28908, Pierce) for 9min and neutralized with glycine. Chromatin was sonicated using a Covaris S2 Focused Ultrasonicator for 8min to achieve a size range of 200–1000bp. Immunoprecipitations were performed overnight using 15μg of sheared chromatin and 10μL of antibody recognizing PR (8757, Cell Signaling). Paired-end sequencing was performed on an Illumina HiSeq, and as a control, input DNA from each experimental condition was also sequenced.

Raw FASTQ files were assessed for quality using fastqc and poor-quality bases were trimmed using trimmomatic (CROP:150 LEADING:3 TRAILING:3 SLIDINGWINDOW:4:15 MINLEN:50). Reads were aligned to the human T2T genome (T2T-CHM13v2.0) using bowtie2 and sam files were generated. Read pairs were fixed, sorted, marked for duplicates and sam files were converted to bam files using samtools. Peaks were called using macs3 (-f BAMPE -g 3117292070 -n $sample_name -B --keep-dup auto --scale-to small --SPMR). Differentially bound regions were identified in R Studio using DiffBind and peaks were annotated using ChIPseeker.

### Meningeal, meningioma cell, and meningioma xenograft short-read RNA sequencing and analysis

For RNA extraction from human meningeal samples, tissue was lysed in TRIzol Reagent (15596018, Thermo Scientific), dissociated using a TissueLyserII (QIAGEN) for 3min at 1.5Hz, and centrifuged at 300rcf for 3min. This was followed by two rounds of phase separation in chloroform and centrifugation to isolate aqueous RNA. RNA was precipitated at −80°C with isopropanol, washed twice with 75% ethanol, air-dried, and resuspended in RNase-free water.

RNA was extracted from cultured meningioma cells and xenograft samples using the RNeasy Mini Kit (74106, QIAGEN). For PR overexpression versus knockdown experiments, cells were cultured in triplicate in 10cm plates to 90% confluence and treated with MPA 1μM or vehicle control for 4hrs or 2 days, as described above, prior to RNA extraction. These timepoints were used to distinguish early genes responding to direct PR transcriptional activity from late genes potentially representing both direct and indirect PR targets. Both PR expressing cells and PR knockdown cells were used to identify which genes were upregulated in response to MPA. For xenograft experiments, tumor tissue was dissected and dissociated using a TissueLyserII (QIAGEN) for 3min at 1.5Hz and centrifuged at 300rcf for 5min in RLT lysis buffer prior to extraction, and RNA was purified using the RNeasy MinElute Cleanup Kit (74204, QIAGEN).

Samples underwent paired-end sequenced on a Novaseq X Plus. Raw FASTQ files were processed using the nf-core rnaseq pipeline, and pipeline defaults were used unless otherwise specified. Briefly, reads were quality controlled using fastqc, trimmed using trimgalore (--clip_r1 10 --clip_r2 10), aligned to the human GRCh38 genome using star, and quantified using salmon. Raw counts were imported into R Studio for analyses. Genes with less than 10 counts in samples fewer than the minimum number of replicates were removed. Differentially expressed genes were identified using DeSeq2 and plotted using EnhancedVolcano. Counts were normalized using variance stabilized transformation, gene expression modules were identified using weighted gene co-expression network analysis (WGCNA), ontologies were identified using clusterProfiler, data were visualized using ComplexHeatmap, with collapsing of samples belonging to same treatment condition or DNA methylation group using the collapseRows (method = “Average”) function from WGCNA. Immune cell type enrichment scores were calculated using xCell and TPM normalized counts from xenograft or human meningioma short-read RNA sequencing data.

### Identification of PR target genes

PR target genes were identified using an integrated approach that incorporated ChIP sequencing data from meningioma cells and RNA sequencing data from human meningiomas, meningioma xenografts, and meningioma cells. WGCNA analysis was performed on human meningioma RNA sequencing data, leading to identification of 37 gene expression modules. Hierarchical clustering of human meningioma RNA sequencing data revealed a cluster of samples with high *PGR* and PR protein (PR^high^). Two WGCNA modules that were comprised of 366 and 898 genes (total 1107 genes), respectively, were enriched in PR^high^ samples and were selected for further analysis. The list of potential PR target genes was refined using RNA sequencing data from meningioma xenografts from female mice and meningioma cells. Of the 1107 candidate genes identified from WGCNA modules, those with higher expression in MPA-treated xenografts from female mice (compared with xenografts from female mice treated with vehicle control) and MPA-treated cells expressing PR (compared with MPA-treated cells with CRISPRi suppression of *PGR* and cells expressing PR after treatment with vehicle control) were retained, resulting in 221 genes from xenografts, 293 genes from cells, and an overlapping list of 58 PR target genes that were enriched in PR^high^ human meningiomas, MPA-treated meningioma xenografts, and MPA-treated meningioma cells. Direct and indirect target genes were defined based on the presence or absence, respectively, of peaks from PR ChIP sequencing of meningioma cells, which distinguished the gene remaining in the two WGNCA nodules.

### Spatial transcriptomic sequencing and analysis

Spatial transcriptomic sequencing was performed on FFPE meningiomas samples with RNA DV200% values greater than 50% using the 10x Genomics Visium Spatial kit (1000336, 10x Genomics). Sectioned cores were mounted within capture areas on Visium slides, deparaffinized, stained with H&E, and imaged. Sequencing libraries were prepared according to manufacturer instructions and sequenced on an Illumina Novaseq X. Sequencing was performed with the recommended protocol (read 1: 28 cycles, i7 index read: 10 cycles, i5 index read: 10 cycles, read 2: 91 cycles). FASTQ sequencing files and histology images were processed using the 10x SpaceRanger pipeline and the Visium Human Transcriptome Probe Set v1.0 GRCh38–2024-A. Data were visualized using the 10x Loupe Browser and Seurat R package.

SpaceRanger filtered feature matrices were imported into a Seurat object (arguments min.cells=3, min.features=100) using R and RStudio. The individual count matrices were normalized based on nFeature_RNA count with less than 10% of reads attributed to mitochondrial transcripts. Dimensionality reduction was performed on the normalized filtered feature-barcode matrix using PCA. UMAP analysis and Louvain clustering were performed on the reduced data, followed by marker identification and differential gene expression. Parameters for downstream analysis included a minimum distance metric of 0.2 for UMAP, resolution of 0.2 for Louvain clustering as determined using clustree, and a minimum difference in fraction of detection of 0.25 and a minimum log-fold change of 0.25 for marker identification. Gene set signature scoring for genes associated with progesterone receptor activity was performed using the AddModuleScore function in UCell, which avoids population wide binning of gene expression and allows more uniform comparisons within and across different datasets. The score was visualized as feature plots using SCpubr.

To define biologically informed thresholds for gene expression of progesterone receptor targe genes, a sequential recursive partitioning analysis (RPA) was performed using the R package rpart on a per sample basis. For each sample in the spatial transcriptomics dataset, a gene set score was first computed as the mean normalized expression per transcriptome of the 58 PR target genes. Two rounds of sequential RPA were then applied to identify optimal cutpoints for three genes of interest. Round 1 included geneA (“PGRMC1”), geneB (“RBM39”), geneC (“PGR”) and round 2 included geneA (“PGRMC1”), geneB (“FXR1”), geneC (“PGR”).

With respect to the gene set score, analysis was performed as follows. Primary split (geneA): A regression tree of depth 1 was fit using geneA expression as the predictor and the gene set score as the response variable. The first split threshold was extracted as the cutpoint for geneA. Secondary split (geneB): The analysis was restricted to spots classified as geneA-positive. Within this subset, a second depth-1 regression tree was fit using geneB expression as the predictor, yielding a geneB cutpoint. Tertiary split (geneC): Within spots classified as both geneA-positive and geneB-positive, a third depth-1 regression tree was fit using geneC expression as the predictor to define the geneC cutpoint. Trees were fit using analysis of variance (ANOVA) splitting with parameters maxdepth = 1, minsplit = 25, and cp = 0.001. Parameters were set that if no valid split was identified at any step, the corresponding cutpoint was recorded as missing, and downstream classifications for that branch were not assigned.

Using the sample-specific cutpoints, each spatial transcriptome was classified into mutually exclusive expression states based on binary thresholds for each gene A/B/C. Binary positivity was defined as expression greater than the corresponding cutpoint. Spatial transcriptomes not meeting any of these criteria were labeled in metadata as “other”. These classifications were stored as metadata within each Seurat object for downstream visualization and quantification. All analyses were performed independently for each sample to account for inter-sample variability in expression distribution. Sample-specific cutpoints for geneA, geneB, and geneC were recorded, along with the number of spots assigned to each gated population. For reproducibility, all intermediate data, including cutpoints, gated classifications, and summary statistics were exported for each sample.

Continuous expression of individual genes and gene set scores was visualized using SpatialFeaturePlot using max.cutoff = “q90” to reduce the influence of extreme values and enhance contrast across spatial regions. Gated expression classes were encoded as binary metadata variables and visualized using SpatialFeaturePlot with min.cutoff = 0, max.cutoff = 1, custom color gradients (negative= ‘grey’, positive=’red’). All plots were generated with consistent visualization parameters (pt.size.factor = 1.5, alpha = c(0.5, 1), image.alpha = 0).

### Enzyme-linked immunosorbent assay (ELISA)

ELISA analysis was performed on flash-frozen serum samples from 55 patients with meningioma using kits for progesterone (10867, Abcam), testosterone (10866, Abcam), and 17-beta-Estradiol (108667, Abcam). Samples were diluted 1:10 in PBS and all samples were run in duplicate, the average of which was used for analysis. Samples with a greater than 10% variance between replicates were re-processed to ensure technical fidelity.

### Breast cancer gene expression analysis

Gene expression analyses were performed using the I-SPY2 pretreatment microarray dataset^177,178^. Analyses were restricted to samples with available clinical annotation and further limited to tumors classified as HER2-negative (HER2−). Expression values were extracted for genes of interest (*FXR1*, *PGRMC1*, and *RBM39*), and sample-level clinical annotations, including ER status and PR status, were extracted from the 2022 I-SPY2 clinical trial dataset and aligned to expression data using harmonized sample identifiers. Within this cohort, tumors were stratified into 3 receptor-defined groups based on ER and PR status: ER+/PR+, ER+/PR−, and ER−/PR+. Differential expression across these groups was assessed using the Kruskal-Wallis rank-sum test. Pairwise Wilcoxon tests with Benjamini-Hochberg adjustment were used for *post hoc* comparisons across receptor groups. Analyses were performed in R using dplyr, ggplot2, rstatix, and ggpubr.

### Statistics

All experiments were performed with independent biological replicates and repeated as indicated. Statistics were derived from independent biological replicates, which are indicated in each figure panel or figure legend. Investigators were blinded to conditions during bioinformatic and functional analyses. Cell cultures and mice were randomized to experimental conditions. No clinical, molecular, or cellular data points were excluded from the analyses. Data distributions were analyzed for normality prior to statistical testing. Results were compared using Student’s t-test, ANOVA, Log-rank test, and other statistical approaches as indicated in the figure legends. Statistical significance is shown by asterisks and defined in the figure legends.

## Supplementary Material

Supplementary Files

This is a list of supplementary files associated with this preprint. Click to download.

- CadyNatureEDFig20260510.docx
- CadyNatureSupplementaryTables20250510.xlsx

## Data Availability

Proteomic and immunoprecipitation mass spectrometry data that were generated for this study will be deposited into the MassIVE database (https://massive.ucsf.edu) prior to publication. The meningioma proteogenomic atlas can be accessed and searched at https://raleighlab.shinyapps.io/meningioma-proteogenomic-atlas/. Genomic data that were generated for this study, including meningeal short-read RNA sequencing, meningioma long-read RNA sequencing, meningioma cell CLIP, ChIP, and short-read RNA sequencing, meningioma xenograft short-read RNA sequencing, and meningioma spatial transcriptomic sequencing, have been deposited to the Gene Expression Omnibus (https://www.ncbi.nlm.nih.gov/geo/) under GSE330184, GSE330185, GSE330018, GSE330019, GSE330042, and GSE330319, which have been combined into GSE330391, or the Sequence Read Archive (https://www.ncbi.nlm.nih.gov/sra) under PRJNA1462352. Previously reported meningioma DNA methylation and CNA profiling data, meningioma RNA and ChIP sequencing data, dog meningioma RNA sequencing data, and breast cancer microarray data, were obtained from GSE183656, GSE101638, GSE139652, GSE212666, PRJNA1045298, and GSE194040, and re-analyzed for this study.

## Code availability

The open-source software, tools, and packages used for data analysis in this study are referenced in the methods where applicable and include the 10x Loupe Browser (v8.1), Aggr (v2.0.0), Seurat (v5.3.0), R (v4.4.3), RStudio (v2026.1.0.392), clustree (v0.5.1), harmony (v1.2.3), UCell (v2.11.1), SCpubr (v3.0.0), IGV (v2.16.0), NxClinical (v6.0), Aperio ImageScope (v12.4.3.5008), Photoshop (v26.2.0), Spectronaut (v18.5.231110.55695 for human, v19.2.240905.62635 for dog), sva (v3.54.0), ConcensusClusterPlus (v1.70.0), factoextra, WGCNA (v1.74), ComplexHeatmap (v2.26.0), limma (v3.64.3), EdgeR (v4.4.2), EnhancedVolcano (v1.26.0), ggplot2 (v4.0.1), reshape2 (v1.4.4), dplyr (v1.1.4), org.Hs.eg.db (v3.21.0), clusterProfiler (v4.16.0), enrichplot (v1.28.4), MAGeCK, *glmnet,* the CLIP Tool Kit (CTK) pipeline, UMI-tools (v1.1.5), cutadapt (v4.9), bwa (v0.7.12), bedtools (v2.31.1), UMICollapse, MPAQT (v0.3.0), kallisto (v0.50.1), lima (v2.12.0), isoseq (v4.2.0), minimap2 (v2.28), bambu (v3.4.1), SUPPA2 (v2.3), circlize (v0.4.16), FragPipe (v23.1), SAINTexpress, artMS, MSstats, AlphaFold3 (v3.0.1), Boltz (v2.2.1), Chai-1 (v0.6.1), MIM (v7.3.2). trimmomatic (v0.39), bowtie2 (v2.5.4), samtools (v1.6), macs3 (v3.0.1), DiffBind (v3.18.0), ChIPseeker (v1.44.0), nf-core rnaseq pipeline (v3.22.2), fastqc (v0.12.1), trimgalore (v0.6.10), star (v2.7.10b), salmon (v1.10.3), DeSeq2 (v1.50.2), xCell (https://comphealth.ucsf.edu/app), SpaceRanger (v2.0.0), and SpatialFeaturePlot (sample script for spatial transcriptomic visualization is available at https://github.com/mdm247/spatialPR.git).
